# Air Pollution From Forest and Vegetation Fires in Southeast Asia Disproportionately Impacts the Poor

**DOI:** 10.1029/2021GH000418

**Published:** 2021-09-01

**Authors:** Carly L. Reddington, Luke Conibear, Suzanne Robinson, Christoph Knote, Stephen R. Arnold, Dominick V. Spracklen

**Affiliations:** ^1^ School of Earth and Environment Institute for Climate and Atmospheric Science University of Leeds Leeds UK; ^2^ Model‐Based Environmental Exposure Science Faculty of Medicine University of Augsburg Augsburg Germany

**Keywords:** landscape fires, health impact assessment, open biomass burning, particulate matter, ozone, ambient air pollution

## Abstract

Forest and vegetation fires, used as tools for agriculture and deforestation, are a major source of air pollutants and can cause serious air quality issues in many parts of Asia. Actions to reduce fire may offer considerable, yet largely unrecognized, options for rapid improvements in air quality. In this study, we used a combination of regional and global air quality models and observations to examine the impact of forest and vegetation fires on air quality degradation and public health in Southeast Asia (including Mainland Southeast Asia and south‐eastern China). We found that eliminating fire could substantially improve regional air quality across Southeast Asia by reducing the population exposure to fine particulate matter (PM_2.5_) concentrations by 7% and surface ozone concentrations by 5%. These reductions in PM_2.5_ exposures would yield a considerable public health benefit across the region; averting 59,000 (95% uncertainty interval (95UI): 55,200–62,900) premature deaths annually. Analysis of subnational infant mortality rate data and PM_2.5_ exposure suggested that PM_2.5_ from fires disproportionately impacts poorer populations across Southeast Asia. We identified two key regions in northern Laos and western Myanmar where particularly high levels of poverty coincide with exposure to relatively high levels of PM_2.5_ from fires. Our results show that reducing forest and vegetation fires should be a public health priority for the Southeast Asia region.

## Key Points

- Eliminating forest and vegetation fires could substantially improve regional air quality in Mainland Southeast Asia
- Reducing exposure to particulate and ozone pollution from fires would yield a considerable public health benefit across Southeast Asia
- Particulate air pollution from fires disproportionately impacts poorer populations across Southeast Asia

## 1 Introduction

Forest and vegetation fires, also referred to as open biomass burning, are a major source of particulate matter (PM) (Chen et al., 2017), ozone (Jaffe & Wigder, 2012), and other air pollutants to the atmosphere and can cause serious air quality issues in many parts of East Asia (Bruni Zani et al., 2020; Crippa et al., 2016; Kiely et al., 2020; Koplitz et al., 2016; Lee et al., 2018; Marlier et al., 2012; Reddington et al., 2014). Observations show that emissions from these fires, which include agricultural residue burning and deforestation fires, influence pollutant concentrations in both rural and urban regions (Janjai et al., 2009; Lasko et al., 2018; Nguyen et al., 2019; Pengchai et al., 2009; Tsai et al., 2013; Zhu et al., 2016). Exposure to smoke from fires is associated with adverse health outcomes including morbidity and mortality (de Oliveira Alves et al., 2017; Jacobson et al., 2014; Jayachandran, 2009; H. J. Johnston et al., 2019; Pienkowski et al., 2017; Pongpiachan & Paowa, 2015; Reid et al., 2016; Vajanapoom et al., 2020). Most previous work has focused on the air quality impacts of fires in Indonesia (Equatorial Asia) (Bruni Zani et al., 2020; Crippa et al., 2016; Kiely et al., 2020; Koplitz et al., 2016; Marlier et al., 2012; Reddington et al., 2014) and the Amazon (Butt et al., 2020; Nawaz & Henze, 2020; Reddington et al., 2015). In this study, we focus on the air quality impacts of fires in Mainland Southeast Asia (Myanmar, Thailand, Cambodia, Lao People's Democratic Republic (hereafter Laos), and Vietnam; also referred to as the Indochina Peninsula or Peninsula Southeast Asia) and south‐eastern China, which have been much less studied.

In Southeast Asia, fires are used as a tool for agricultural management, for example, to remove agricultural residues (mainly from rice and sugarcane cultivation) and weeds, and for forest clearance for agricultural purposes (Biswas et al., 2015; Chen et al., 2017; Phairuang et al., 2017). Fires in Mainland Southeast Asia mainly occur during the premonsoon season (roughly February to April), due to widespread forest fires and crop residue burning in preparation for planting at the Asian summer monsoon onset (W.‐R. Huang et al., 2016; Phairuang et al., 2017). The increased fire activity coincides with a widespread stable temperature inversion layer over Thailand, Vietnam, Laos, and Southern China (Nodzu et al., 2006) with hot, dry, and stagnant air over northern Thailand (Kim Oanh & Leelasakultum, 2011) promoting haze conditions. During the burning season, long‐range transport of smoke from fires in Mainland Southeast Asia has been observed in Southwest China (Zhu et al., 2016), south‐eastern Tibetan Plateau (Sang et al., 2013), Southern China, Taiwan, and Hong Kong (K. Huang et al., 2013). Fires reduce substantially after the onset of the summer monsoon rainfall (in late April) and are minimal until around the start of the dry season (in November). Fires in this region display a degree of interannual variability linked to changes in atmospheric circulation features, such as the India‐Burma Trough (W.‐R. Huang et al., 2016).

Several studies have used a mix of models and observations to explore the impacts of fire on atmospheric aerosol properties, visibility, and/or air quality in Mainland Southeast Asia (Duc et al., 2016; K. Huang et al., 2013; Lee et al., 2017, 2018; Li et al., 2017; Lin et al., 2013; Vongruang & Pimonsree, 2020; Yin et al., 2019). However, studies quantifying the contribution of fire to particulate air pollution, population exposure and public health are lacking in this region (H. J. Johnston et al., 2019; Yadav et al., 2017), compared in particular to the large number of studies focused on Equatorial Asia (e.g., Crippa et al., 2016; Kiely et al., 2020; Koplitz et al., 2016; Marlier et al., 2012). Recent studies show that fire is the dominant cause of the variation of local ambient air quality in Mainland Southeast Asia (Yin et al., 2019); contributing 49% of ambient PM_10_ (particulate matter with aerodynamic diameter ≤ 10 μm) concentrations during peak open burning in March 2012 (Vongruang & Pimonsree, 2020) and 70%–80% to aerosol optical depth in source regions during March‐April 2013 (Li et al., 2017). Preventing fire could yield substantial reductions in population‐weighted PM_2.5_ (particulate matter with aerodynamic diameter ≤ 2.5 μm) concentrations across Mainland Southeast Asia (Reddington, Conibear, et al., 2019). There are large uncertainties associated with quantifying and simulating particulate emissions from fire in tropical regions (Reddington et al., 2016). In Mainland Southeast Asia, there is a large range in emissions estimates (Kaiser et al., 2012; Lasko et al., 2017; Shi & Yamaguchi, 2014; Sornpoon et al., 2014; Phairuang et al., 2017; van der Werf et al., 2017; Wiedinmyer et al., 2011) and varying performance when tested in models against observations (Fu et al., 2012; Lee et al., 2017; Pimonsree et al., 2018; Reddington et al., 2014, 2016; Takami et al., 2020; Vongurang et al., 2017). Emissions from the Fire Inventory from NCAR (FINN; Wiedinmyer et al., 2011) have been used widely in models over this region; with simulated PM concentrations showing good agreement against observations in some studies (Reddington et al., 2014, 2016; Takami et al., 2020), but overestimation by a factor of ∼2 in others (Li et al., 2017; Pimonsree et al., 2018; Vongurang et al., 2017). Fires also impact ozone concentrations, being a source of ozone precursors and altering photochemistry, impacting ozone production (Jaffe & Wigder, 2012). The efficacy of photochemical ozone production in fire plumes is highly variable and uncertain, and is affected by nonlinear ozone dependence on changes in precursor concentrations, and high particulate loadings, which affect photochemistry (Jaffe & Wigder, 2012). Fires have been shown to enhance regional ozone concentrations in Mainland Southeast Asia (Pochanart et al., 2001) and aloft over southern China (C. Y. Chan et al., 2003; L. Y. Chan et al., 2000; Kondo et al., 2004), although fires have also been implicated in suppressed ozone in some situations (Deng et al., 2008).

Links between socioeconomic factors, population exposure to ambient air pollution, and associated health effects have been well documented in parts of North America and Europe (e.g., Fairburn et al., 2019; Hajat et al., 2015). However, few studies have focused on countries in Southeast Asia, with some demonstrating strong connections between ambient air pollution and poverty, for example, in urban areas of Laos (S. Dasgupta et al., 2005), rural areas of Vietnam (Narloch & Bangalore, 2018) and Ho Chi Minh City (Mehta et al., 2014); and others finding only weak connections, for example, in Cambodia and Vietnam (S. Dasgupta et al., 2005) or no connection, for example, in Laos (Pasanen et al., 2017). The majority of these studies explored links between poverty and multiple environmental risks, including ambient air pollution from all sources. To our knowledge, no previous studies have examined the poverty levels of populations exposed to air pollution from fires in this region.

In this work, we use a combination of satellite‐derived data sets of fire emissions, models and observations to quantify the contribution of forest and vegetation fires to air quality degradation and disease burden in Mainland Southeast Asia and south‐eastern China. We also examine the poverty levels of the Southeast Asian population exposed to PM_2.5_ pollution derived specifically from fire emissions.

## 2 Materials and Methods

### 2.1 Description of the GLOMAP Global Aerosol Model

We used the Global Model of Aerosol Processes (GLOMAP; Mann et al., 2010; Spracklen et al., 2005) to simulate multiyear (2003–2015) PM concentrations and evaluate the performance of three fire emissions data sets against observations. Table 1 summarizes the model setup used for this study; see Section S1.1 and Reddington et al. (2016) and Reddington, Morgan, et al. (2019) for further details.

**Table 1:** Summary of the Model Setups for the GLOMAP Global Model and WRF‐Chem Regional Model

Model	GLOMAP (v7)	WRF‐Chem (v3.7.1)
Domain	Global	Regional: East Asia
Horizontal resolution	2.8° × 2.8°	30 × 30 km (∼0.3° × 0.3°)
Vertical levels	30 (up to 10 hPa)	33 (up to 10 hPa)
Anthropogenic emissions	MACCity (Granier et al., 2011) for 2003–2010	EDGAR‐HTAP2 (Janssens‐Maenhout et al., 2015) for 2010
Fire emissions	FINN1.5, GFAS1.2, GFED4	FINN1.5
Meteorology	Driven by ECMWF fields	Nudged to NCEP GFS fields (NCEP, 2000, 2007)
Aerosol size distribution	Modal scheme (7 log‐normal modes)	Sectional scheme (MOSAIC 4‐bin; Zaveri et al., 2008)
Gas‐phase chemistry	TOMCAT (Chipperfield, 2006)	MOZART‐4 (Emmons et al., 2010)
Simulation year(s)	2003–2015	2014
Simulations	(1) GLOMAP_nofire: fire emissions excluded	(1) WRFChem_nofire: fire emissions excluded
	(2) GLOMAP_FINN: with FINN fire emissions	(2) WRFChem_FINN: FINN fire emissions
	(3) GLOMAP_GFAS: with GFAS fire emissions	(3) WRFChem_FINNx1.5: FINN fire emissions scaled upwards by a factor 1.5
	(4) GLOMAP_GFED: with GFED fire emissions	

Abbreviations: GLOMAP, Global Model of Aerosol Processes; WRF‐Chem, Weather Research and Forecasting model coupled with Chemistry.

#### 2.1.1 Fire Emissions in GLOMAP

Fire emissions of sulfur dioxide (SO_2_), black carbon (BC), and organic carbon (OC) were specified using three different data sets: the National Center for Atmospheric Research Fire Inventory version 1.5 (FINNv1.5) (Wiedinmyer et al., 2011), the Global Fire Emissions Data Set version 4.1 with small fires (GFED4s) (van der Werf et al., 2010, 2017) and the Global Fire Assimilation System versions 1.0 and 1.2 (GFASv1.0 and GFASv1.2) (Kaiser et al., 2012); hereafter referred to as FINN, GFED, and GFAS, respectively. The different fire emission estimation methodologies of these data sets are described in detail in their references given above and in our previous work (Reddington et al., 2016; Reddington, Morgan, et al., 2019). We use daily fire emissions from all three data sets (daily GFED emissions are available from 2003 onwards [Mu et al., 2011]). Fire emissions were distributed vertically over six ecosystem‐dependent altitudes between the surface and 6 km according to Dentener et al. (2006). Over Mainland Southeast Asia, all emissions were injected below 3 km elevation, which is consistent with satellite observations of the vertical distribution of smoke in this region (Gautam et al., 2013).

#### 2.1.2 GLOMAP Model Simulations

We performed four model simulations with GLOMAP: one simulation excluding fire emissions (“GLOMAP_nofire”); and three simulations each including a different fire emissions data set (“GLOMAP_FINN”, “GLOMAP_GFED,” and “GLOMAP_GFAS”). Simulations were run from January 1, 2003 to December 31, 2015 (after a 92‐day spin‐up), driven by ECMWF ERA‐Interim global reanalyzes (Dee et al., 2011) that correspond to the simulation date/time.

### 2.2 Description of the WRF‐Chem Regional Model

We used the Weather Research and Forecasting model coupled with Chemistry (WRF‐Chem; Grell et al., 2005) version 3.7.1, a high‐resolution regional model, to simulate air pollutant concentrations for 1 year (2014) and quantify the public health impacts of long‐term exposure to fire‐derived PM_2.5_ and ozone (O_3_) concentrations. Table 1 summarizes the model setup used for this study; see Section S1.2 for further details.

#### 2.2.1 Fire Emissions in WRF‐Chem

Fire emissions were taken from FINN version 1.5 (Wiedinmyer et al., 2011), with a spatial resolution of 1 × 1 km for the year 2014. Fire emissions were included for BC, OC, PM_2.5_, PM_10_, carbon monoxide, ammonia, nitrogen oxides, SO_2_, and nonmethane volatile organic compounds (speciated according to the Model for Ozone and Related Chemical Tracers (MOZART); Emmons et al., 2010). We applied a diurnal factor (Western Regional Air Partnership, 2005) to the daily emissions, which assumes greater emissions during the day (between 10:00 and 19:00 local time, peaking at 15:00–16:00 local time) and minimal emissions during the night. The injection heights of the fire emissions were calculated online in the model using the Freitas et al. (2007) plume‐rise parameterization. The plume‐rise parameterization applies a 1‐D cloud‐parcel model to each grid‐column within the WRF‐Chem model domain that contains a fire.

#### 2.2.2 WRF‐Chem Model Simulations

The model domain is located over East Asia, using a Lambert conformal conical projection with a horizontal resolution of 30 × 30 km (covering a 130 × 124 grid) and 33 vertical levels up to a minimum pressure of 10 hPa. We regridded the model output, using linear interpolation, onto a regular latitude‐longitude grid at 0.25° × 0.25° resolution. We performed three model simulations with WRF‐Chem: one simulation excluding fire emissions (“WRChem_nofire”); one simulation including FINN fire emissions (“WRFChem_FINN”); and one simulation where FINN fire emissions of OC and BC were scaled upwards by a factor 1.5 (“WRFChem_FINNx1.5”). The simulation period was for 1 year from January 9, 2014 to January 9, 2015, with the first 8 days of January 2014 run as spin‐up. We selected 2014 for our simulation year since both PM and O_3_ measurements are available for this year (Section 2.5).

### 2.3 Description of the Public Health Impact Assessment

We estimated the disease burden attributable to ambient PM_2.5_ exposure (simulated by WRF‐Chem) using population attributable fractions of relative risk. The relative risk of disease at a specific ambient PM_2.5_ exposure was estimated through the Global Exposure Mortality Model (GEMM) (Burnett et al., 2018). We calculated the disease burden due to long‐term exposure to ambient O_3_ (simulated by WRF‐Chem) using the exposure‐response function from Turner et al. (2016). Uncertainty intervals at the 95% confidence level (95UI) were estimated through using the derived uncertainty intervals from the exposure‐outcome functions, baseline mortality and morbidity rates, and population age fractions. See Section S1.3 for further details.

The mortality due to fire emissions (*M*_FIRE_) was calculated using the “subtraction” method (Conibear et al., 2018); calculating the difference between the premature mortality from all sources (*M*_ALL_) and the premature mortality when fire emissions have been removed (*M*_FIRE_OFF_) as in Equation 1:

(1) $$M_{\text{FIRE}}=M_{\text{ALL}}–M_{\text{FIRE\_OFF}}$$

### 2.4 Description of the Poverty Proxy Data

As a proxy for population poverty levels, we used gridded subnational Infant Mortality Rate (IMR) estimates from NASA Socioeconomic Data and Applications Center for 2015 (Center for International Earth Science Information Network (CIESIN), 2018b). For further details see Section S1.5 and Figure S8.

The IMR is defined as the number of children who die before their first birthday for every 1,000 live births in a given year. For context, previous studies have defined populations with IMR < 15 to be not poor; 15 ≤ IMR < 32 to be moderately poor; 32 ≤ IMR < 65 to be poor; and 65 ≤ IMR < 100 to be very poor (De Sherbinin, 2008); and populations with a high IMR as having > 32 deaths per 1,000 live births (Barbier & Hochard, 2019).

Subnational IMR estimates have been used as a proxy for poverty indicators in a range of previous studies (Barbier & Hochard, 2018, 2019; Barlow et al., 2016; De Sherbinin, 2008; Hauenstein et al., 2019). A strong correlation between IMR and other poverty‐related metrics, including population income, education and health (De Sherbinin, 2008; Fritzell et al., 2015; O'Hare et al., 2013; Reidpath & Allotey, 2003; Sartorius & Sartorius, 2014), justifies the use of IMR as a proxy for overall poverty levels. In addition, it is difficult to obtain alternative poverty measures at sub‐national levels for multiple countries (CIESIN, 2018a; P. Dasgupta, 1993). Other advantages of this data set over alternative poverty measures include its highly standardized nature and availability for ≥90% of medium‐income and low‐income country populations (Balk et al., 2006; CIESIN, 2018a).

### 2.5 Description of the Particulate Matter and Ozone Measurements

We used 2003–2015 monthly mean PM_10_ concentrations measured at air quality monitoring stations located in fire‐influenced regions of Thailand (Figure S1a) from the Pollution Control Department (PCD) of the Thailand Government Ministry of Natural Resources and Environment. The fire‐influenced stations were selected using GLOMAP or WRF‐Chem model data where fire emissions contributed 20% or greater to the simulated annual mean PM_10_. We used surface O_3_ concentration measurements from air quality monitoring stations located in China and surrounding countries (Figure S1b) from the Berkley Earth China Air Quality Data Set (Rohde & Muller, 2015). See Section S1.4 for further details on the measurements.

To evaluate model‐simulated surface PM_10_ concentrations due only to the influence of fire, we calculated and compared simulated and measured fire‐derived (smoke) PM_10_ concentrations. The simulated and measured fire‐derived PM_10_ concentrations were estimated for each year separately, by subtracting the minimum monthly mean PM_10_ concentration from all monthly mean concentrations for that year. A similar approach has been used in previous modeling studies (e.g., Kiely et al., 2020) to isolate enhancements in surface PM concentrations due only to fires.

To quantify the agreement between model and observations, we used the Pearson correlation coefficient (*r*) and normalized mean bias factor (NMBF) as defined by Yu et al. (2006). A positive NMBF indicates the model overestimates the observations by a factor of NMBF + 1. A negative NMBF indicates the model underestimates the observations by a factor of 1 – NMBF.

## 3 Results

### 3.1 Analysis of Fire Emissions Over Southeast Asia

**Figure 1:**
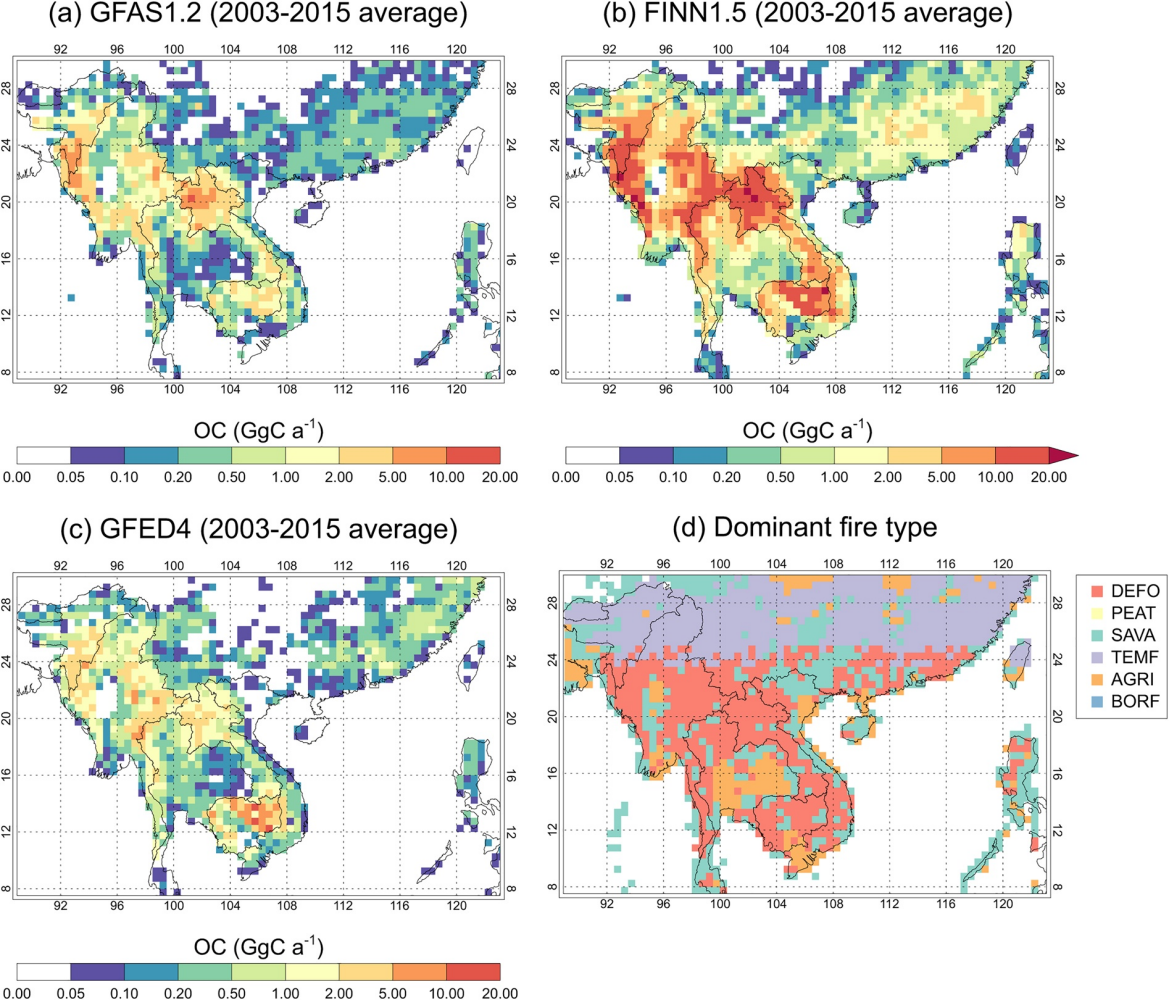
(a–c) Annual total organic carbon (OC) emissions from fire across Southeast Asia, averaged over the period (2003–2015) from three fire emission data sets: (a) GFAS version 1.0 (and version 1.2 from 2012 onwards), (b) FINN version 1.5, and (c) GFED version 4.1s (GFED4). Fire emissions are all re‐gridded to 0.5° × 0.5° resolution for comparison. (d) Spatial distribution of the dominant fire types for fire emissions of OC for 2003–2015. Data are from GFED4 (van der Werf et al., 2010) regridded to 0.5° × 0.5° resolution. Fires are characterized into six types: Deforestation and degradation fires (DEFO); Peatland fires (PEAT); Savanna, grassland, and shrubland fires (SAVA); Temperate forest fires (TEMF); Agricultural waste burning (AGRI); and Boreal forest fires (BORF). The dominant fire type was derived by calculating the maximum GFED4 OC emissions flux for each fire type in each 0.5° × 0.5° grid cell over the period 2003–2015.

Figure 1 shows the 2003–2015 average spatial distribution of OC emissions from fire over Southeast Asia from GFAS, FINN, and GFED. In all data sets, greatest emissions occur in the northern regions of Laos, Cambodia, and Thailand, eastern and western Myanmar and southern Bangladesh, and lower emissions in central regions of Myanmar and Thailand, northern Vietnam and south‐eastern China. The regions of greatest OC emissions are dominated by deforestation and degradation fires (as classified by GFED4; van der Werf et al., 2017, Figure 1d). FINN generally estimates greatest OC emissions of the three emission data sets across the region, with lowest OC emissions estimated by GFED.

**Figure 2:**
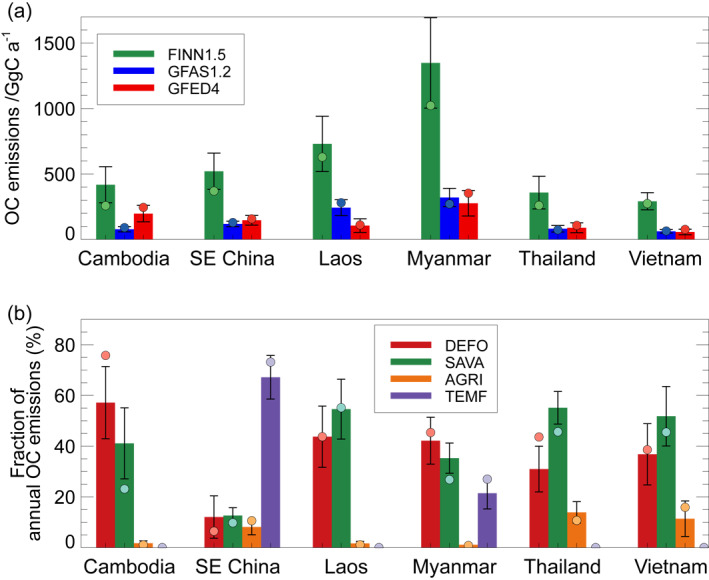
(a) Annual total organic carbon (OC) emissions from fire for countries/regions in Southeast Asia. Bars show annual total emissions averaged over the period (2003–2015) with error bars showing the standard deviation; circles show annual total emissions for 2014. OC emissions are shown from three fire emission data sets: GFAS version 1.0 (and version 1.2 from 2012 onwards), FINN version 1.5 and GFED version 4.1s (GFED4). “SE China” is defined as south of 30°N and east of 98°W. (b) Fire type fraction of GFED4 annual total OC emissions for four different fire types: Deforestation and degradation fires (DEFO); Savanna, grassland, and shrubland fires (SAVA); Agricultural waste burning (AGRI); and Temperate forest fires (TEMF) (van der Werf et al., 2010). Bars show fire type fractions averaged over the period (2003–2015) with error bars showing the standard deviation; circles show fire type fractions for 2014.

Figure 2 shows the 2003–2015 average annual OC emissions at the country scale with the greatest emissions from Myanmar and lowest from Vietnam. Countrywide FINN OC emissions are a factor 2–7 greater than GFED and a factor 3–5 greater than GFAS. Annual OC emissions summed across the region vary by a factor of 4 (GFAS: 0.90 Tg a^‐1^; FINN: 3.67 Tg a^‐1^; GFED: 0.87 Tg a^‐1^) and contribute between 5% (GFAS) and 18% (FINN) of 2003–2015 average global fire OC emissions. The importance of particulate fire emissions in this region depends on the fire emissions data set used. In the FINN data set, domain‐wide fire OC emissions (3.7 Tg a^‐1^) are comparable to long‐term average annual fire OC emissions in northern South America (3.1 Tg a^‐1^; Butt et al., 2020).

Differences in the magnitude of OC emissions estimated by the three data sets arise from multiple factors involved in the different fire detection and emission estimation methods used, for example, differences in the land use/land cover classifications used and the emissions factors assumed for various fire types and aerosol species (T. Liu et al., 2020); and possible biases in regions of agricultural residue burning and small savanna/grassland fires (Randerson et al., 2012; T. Zhang, Wooster, et al., 2018).

Across Mainland Southeast Asia, fire emissions are predominantly from deforestation/degradation fires (accounting for 31%–57%) and savanna‐type fires (accounting for 35%–55%) (Figure 2b). A detailed analysis of forest fires in Myanmar confirms that most are of anthropogenic origin (Biswas et al., 2015). Vadrevu et al. (2019) found that most fires occurred in forests as opposed to cropland across much of Mainland Southeast Asia including Myanmar, Laos, Cambodia, and Vietnam. In regions with both deforestation and savanna fires, deforestation fires emit a greater amount of particulate emissions, due to a combination of larger fuel loads/biomass consumption and emission factors, and thus tend to dominate emissions (Figure 1d). However, savanna fires are more prevalent across the region and so the accumulated emissions from this fire type per country are generally comparable to or greater than deforestation fires. In south‐eastern China, OC emissions arise predominantly from fires classified as temperate forest fires (67%). Agricultural fires make up a relatively small fraction of fire OC emissions across the region (1%–14%), but the occurrence of these fires may be underestimated or misrepresented both in GFED (Reddington et al., 2016; T. Zhang, Wooster, et al., 2018), and more widely by satellite‐based estimates (Lasko et al., 2017; Shen et al., 2019; Stavrakou et al., 2016; L. Zhang et al., 2016; T. Zhang et al., 2020).

### 3.2 Model Evaluation

#### 3.2.1 Evaluation of Fire Emissions Data Sets

**Figure 3:**
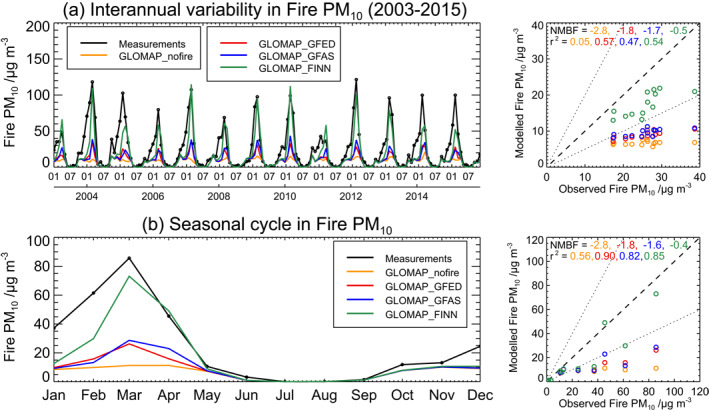
Evaluation of GLOMAP‐simulated PM_10_ over Thailand. (a) Left: time‐series of simulated and measured monthly mean fire‐derived PM_10_ concentrations between 2003 and 2015, averaged over 12 fire‐influenced stations (shown in Figure S1a); Right: simulated versus measured annual mean fire‐derived PM_10_. (b) Left: time‐series of simulated and measured multiannual average seasonal cycle of fire‐derived PM_10_ concentrations, averaged over the same stations as the upper panel; Right: simulated versus measured multiannual monthly mean fire‐derived PM_10_. The model bias (NMBF) and correlation (*r* ^2^) between modeled and measured values are given at the top of the righthand figures. Simulated concentrations are shown for the model with FINN1.5 (GLOMAP_FINN), GFAS1.2 (GLOMAP_GFAS), GFED4 (GLOMAP_GFED) emissions, and without fire emissions (GLOMAP_nofire).

Figure 3 compares three fire emissions data sets in GLOMAP against long‐term surface measurements of PM_10_ from 12 fire‐influenced stations in Thailand. The measurements show a consistent peak in monthly mean fire‐derived PM_10_ concentrations of ∼60–130 mg m^−3^ during the premonsoon season (roughly between January and May) across all years. Annual peak concentrations show a moderate degree of interannual variability, with relatively low peaks measured during 2003, 2008 and 2011 (and relatively high in 2004, 2007, and 2012). The multiyear GLOMAP simulations demonstrate that fires consistently make a substantial contribution to surface PM_10_ concentrations in northern Thailand over a 13‐year period.

Figure 3a shows GLOMAP generally captures the measured interannual variability in fire‐derived PM_10_ when fire emissions are included in the model (*r* ^2^ = 0.47–0.57, depending on the emission data set) but underestimates the magnitude of the measurements in all simulations (NMBF = −1.8 to −0.5), particularly in 2005, 2014, and 2015. The smallest model bias in annual mean fire‐derived PM_10_ across all years (NMBF = −0.5) is achieved with FINN emissions.

Figure 3b shows the strong seasonal variability in measured fire‐derived PM_10_ concentrations, with average concentrations peaking in March and then decreasing to very low values between May and September. The measured seasonal variation is captured well in the simulations with fire emissions (*r* ^2^ = 0.82–0.90, depending on the emission data set). However, the magnitude of fire‐derived PM_10_ concentrations is best captured by the model with FINN emissions (Figure 3b; NMBF = −0.4; see further analysis in Section S2.1 and Figure S2). This result is consistent with our previous work (Reddington et al., 2016) that used AERONET aerosol optical depth to evaluate the GLOMAP model over Southeast Asia. Therefore, we use the FINN emissions in our high‐resolution regional model simulations in the following sections.

#### 3.2.2 Evaluation of WRF‐Chem Particulate Matter Concentrations

**Figure 4:**
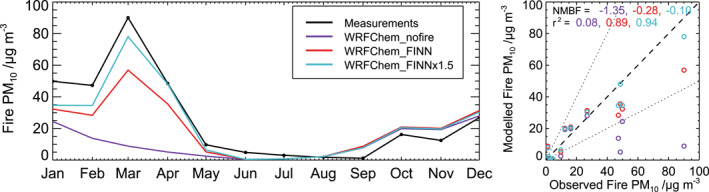
Evaluation of Weather Research and Forecasting model coupled with Chemistry (WRF‐Chem)‐simulated PM_10_ over Thailand. Left: Time‐series of simulated and measured monthly mean fire‐derived PM_10_ concentrations during 2014 averaged over 12 fire‐influenced stations (shown in Figure S1a). Right: simulated versus measured annual mean fire‐derived PM_10_. The model bias (NMBF) and correlation (*r* ^2^) between modeled and measured values are given at the top of the righthand figure. Simulated concentrations are shown for the model without fire emissions (WRFChem_nofire), and for the model with FINN emissions (WRFChem_FINN) and with FINN emissions scaled upwards by a factor 1.5 (WRFChem_FINNx1.5).

Figure 4 compares WRF‐Chem simulated and measured regional‐average seasonal cycles in fire‐derived PM_10_ for 12 fire‐influenced stations in Thailand during 2014. We note that annual fire emissions in FINN for 2014 are comparable to or lower than the 2003–2015 average (Figure 2a). The model with FINN emissions well simulates the monthly mean variation in measured fire‐derived PM_10_ concentrations (*r* ^2^ = 0.89) but underestimates the magnitude of the observations (NMBF = −0.28) predominantly during January to July. This is consistent with *total* PM_10_ concentrations (Figure S3).

Increasing the particulate fire emissions by a factor 1.5 improves the overall agreement with measured fire‐derived PM_10_ (Figure 4; *r* ^2^ = 0.94, NMBF = −0.10). Specifically, the FINNx1.5 simulation better captures the measured seasonal variation and magnitude of fire‐derived PM_10_ at 11 out of 12 stations (Figure S4; FINN: normalized standard deviation (NSD) = 0.55–1.21; FINNx1.5: NSD = 0.64–1.74), with little change in the strong temporal correlation (FINN: *r* = 0.83–0.97; FINNx1.5: *r* = 0.87–0.98). The FINNx1.5 simulation also agrees well with PM_2.5_ measurements (see Section S2.2 and Figure S5). Previous studies have used similar or larger scaling factors to increase fire emissions in models to better match observations (see Reddington et al., 2016 and references therein). In the following sections, we show results from the FINNx1.5 simulation as it gives the best match to PM observations.

#### 3.2.3 Evaluation of WRF‐Chem Surface Ozone Concentrations

**Figure 5:**
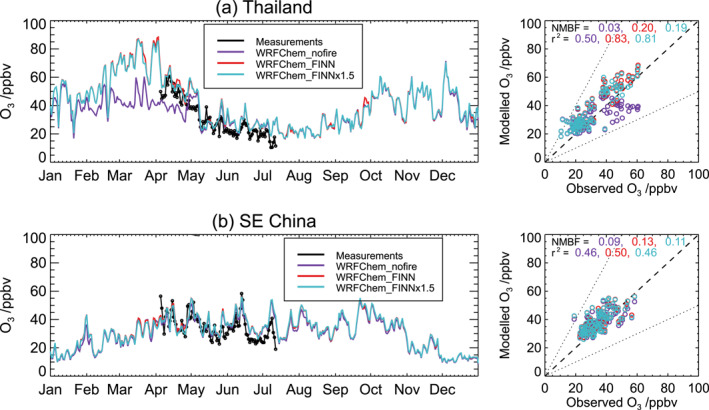
Evaluation of Weather Research and Forecasting model coupled with Chemistry (WRF‐Chem)‐simulated ozone (O_3_) over Thailand and South‐eastern (SE) China. Left: Time‐series of simulated and measured daily mean surface O_3_ mixing ratios during 2014; Right: simulated versus measured daily mean O_3_. Regional averages are shown for: (a) Thailand (9 air quality monitoring stations); and (b) SE China (368 stations in south‐eastern Mainland China, 72 stations in Taiwan/Republic of China, and 12 stations in Hong Kong Special Administrative Region). O_3_ measurements are available from April–July 2014. The model bias (NMBF) and correlation (*r* ^2^) between modeled and measured values are given at the top of the righthand figures. Simulated values are shown for three model simulations: without fire emissions (WRFChem_nofire); with FINN fire emissions (WRFChem_FINN); and with FINN emissions scaled upwards by a factor 1.5 (WRFChem_FINNx1.5).

Figure 5 compares simulated and measured daily mean surface O_3_ mixing ratios averaged over two regions in Southeast Asia during April–July 2014. Regional‐average measured O_3_ mixing ratios range from ∼10 to ∼60 ppbv. Variability in surface O_3_ concentrations over Southeast Asia is driven by a complex mix of factors, including varying precursor gas emissions and concentrations, photochemical production, and meteorological effects (causing accumulation, transport and removal). We evaluate the model against total O_3_ rather than fire‐derived O_3_, as for total PM_2.5_ in Section S2.2, because these quantities are used for the health impact assessment in Section 3.4.

Measured surface O_3_ mixing ratios in Thailand show a peak during April (Figure 5a), which has been reported to be due to regional scale O_3_ production triggered by fires (Chen et al., 2017; Pochanart et al., 2001). The FINNx1.5 simulation captures this peak and reproduces the general daily variability in measured O_3_ concentrations (*r* ^2^ = 0.81), while slightly overestimating the magnitude of the measurements (NMBF = 0.19). In south‐eastern China (Figure 5b), the model simulates the magnitude and temporal variability of the measured O_3_ mixing ratios reasonably well (*r* ^2^ = 0.46, NMBF = 0.11). Model‐measurement comparisons are shown for separate provinces/regions in south‐eastern China in Figure S6. Previous studies have reported increased ozone concentrations aloft (∼2–6 km altitude) over southern China due to fires in Mainland Southeast Asia but show little enhancement at the surface (C. Y. Chan et al., 2003; L. Y. Chan et al., 2000; Kondo et al., 2004), consistent with the model results. Reductions in photochemical ozone production as a result of PM from fires can also act to reduce ozone concentrations (Deng et al., 2008).

### 3.3 Impacts of Forest and Vegetation Fires on Air Quality

**Figure 6:**
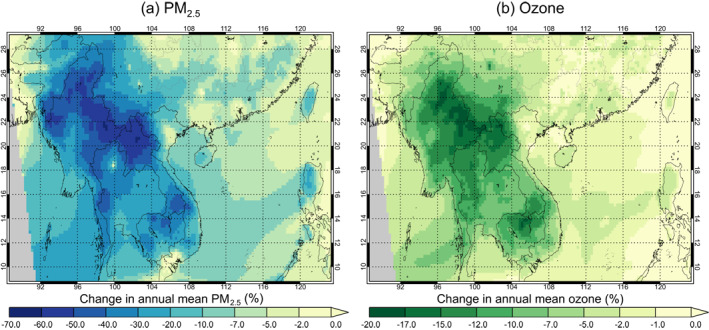
The air quality effects of eliminating fire across Southeast Asia. Shown are the percentage changes in Weather Research and Forecasting model coupled with Chemistry (WRF‐Chem)‐simulated annual (2014) mean (a) PM_2.5_ and (b) ozone concentrations at ground level when fire emissions are excluded in the model. Results are shown for the high fire emissions scenario (WRFChem_FINNx1.5). Regions in gray are outside the model domain.

Figure 6a shows the relative change in simulated surface annual (2014) mean PM_2.5_ concentration when fire emissions are excluded in WRF‐Chem (see Figure S7 for simulated annual mean surface concentrations). Eliminating fire emissions reduces simulated annual mean surface PM_2.5_ concentrations by ∼40%–70% in northern Thailand, Myanmar, Cambodia, and Laos, with reductions in south‐eastern China ranging from ∼10%to 40% in the region of Mainland Southeast Asia and in Taiwan, to ≤10% in the provinces further east. Population‐weighted annual mean PM_2.5_ concentrations across Southeast Asia are reduced by 7%, with reductions of 20% in Cambodia, 41% in Laos, 31% in Myanmar, 23% in Thailand, and 7% in Vietnam.

Simulated PM_2.5_ concentrations suggest that for 2014, the World Health Organization (WHO) Air Quality Guideline for PM_2.5_ (an annual mean of 10 μg m^−3^; WHO, 2006) is exceeded in almost every location in Southeast Asia even when fires are excluded (see Figures S7a and S7b). However, excluding fires substantially reduces the population exposed to levels of PM_2.5_ above the WHO Air Quality Interim Target 2 (annual mean of 25 μg m^−3^) in Thailand (by 64%), Myanmar (by 100%), Laos (by 92%), and Cambodia (by 44%), with smaller reductions in Vietnam (by 9%) and south‐eastern China (by 3%).

Figure 6b shows the relative change in simulated surface annual mean O_3_ concentration when fire emissions are excluded from the model (see Figures S7c and S7d for absolute concentrations). The spatial pattern of relative changes in surface O_3_ is fairly consistent with the peak and minimum relative changes in surface PM_2.5_ concentrations, with largest reductions over northern Thailand, Myanmar, Cambodia, and Laos (up to 20%) and smaller reductions over most of south‐eastern China (<15%). When fires are excluded from the model, the annual average daily maximum 8‐h (ADM8h) O_3_ concentration is reduced by 5% across Southeast Asia, with reductions of 10% in Cambodia, 12% in Myanmar and Laos, 8% in Thailand, 5% in Vietnam, and 2% in south‐eastern China.

### 3.4 Impacts of Forest and Vegetation Fires on Public Health

Table 2 shows the averted disease burden due to changes in long‐term exposure to ambient PM_2.5_ and O_3_ from eliminating fire emissions. Eliminating fire emissions reduces the annual disease burden from ambient PM_2.5_ exposure by 12% in Mainland Southeast Asia (ranging from 5% in Vietnam to 28% in Laos), averting a total of 27,500 (95UI: 24,700–30,400) premature deaths. In south‐eastern China, the disease burden is reduced by 3%, averting 31,400 (95UI: 30,500–32,400) premature deaths. Assuming a low fire scenario (FINN) decreases the averted annual PM_2.5_ disease burden from eliminating fire emissions by a factor of 1.3 (Table S2).

**Table 2:** Averted Public Health Effects due to Changes in Long‐Term Exposure to Ambient PM_2.5_ and Ozone (O_3_) From Eliminating Fire Emissions

Country/region	Reduction in PM_2.5_ exposure	Reduction in PM_2.5_ MORT	PM_2.5_ MORT (yr^−1^)	PM_2.5_ DALYs (yr^−1^)	Reduction in O_3_ exposure	Reduction in O_3_ MORT	O_3_ MORT (yr^−1^)
Cambodia	20%	13%	1,500 (1,300–1,700)	59,500 (49,100–71,700)	10%	15%	140 (130–160)
Laos	41%	28%	1,600 (1,300–1,800)	63,600 (49,400–77,400)	12%	16%	80 (70–80)
Myanmar	31%	21%	10,800 (9,500–12,000)	393,100 (326,200–467,300)	12%	20%	1,070 (940–1,190)
Thailand	23%	15%	8,500 (7,900–9,100)	344,500 (288,600–405,700)	8%	7%	600 (550–650)
Vietnam	7%	5%	5,100 (4,600–5,700)	186,800 (145,100–225,400)	5%	4%	360 (310–390)
SE China	5%	3%	31,400 (30,500–32,400)	1,042,900 (919,200–1,184,800)	2%	1%	1,530 (1,380–1,660)
Total Mainland SE Asia	16%	12%	27,500 (24,700–30,400)	1,047,500 (867,500–1,247,300)	9%	10%	2,250 (2,000–2,470)
Total SE Asia domain	7%	5%	59,000 (55,200–62,900	2,090,300 (1,786,700–2,432,200)	5%	3%	3,790 (3,380–4,130)

*Note*. Shown are the percentage reductions in population‐weighted annual mean PM_2.5_ concentration (PM_2.5_ exposure), annual mean daily maximum 8‐h (ADM8h) O_3_ concentration (O_3_ exposure), and annual disease burden; and the numbers of averted annual premature mortalities (MORT) and disability‐adjusted life years (DALYs) per country for the higher fire emissions scenario (FINNx1.5). Values in parentheses represent the 95% uncertainty intervals (95UI). PM_2.5_ mortality values are rounded to the nearest 100 and O_3_ mortality values are rounded to the nearest 10. “SE China” is defined as south of 30°N and east of 98°W, and includes Hong Kong SAR, Macau SAR and Taiwan. “Mainland SE Asia” includes Cambodia, Laos, Myanmar, Thailand, and Vietnam

**Figure 7:**
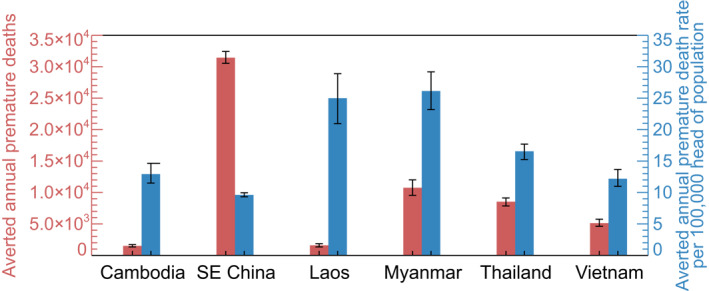
The number of averted annual premature mortalities across Southeast Asia due to changes in long‐term exposure to ambient PM_2.5_ from eliminating fire emissions. The total annual premature mortality estimates are shown for each country by the red bars; the annual premature mortality rate estimates (mortalities per 100,000 head of population) are shown for each country by the blue bars. Error bars represent the 95% uncertainty intervals.

Figure 7 shows the averted annual premature mortalities and mortality rate by country from eliminating fire emissions. While the number of avoided total premature mortalities is much higher in south‐eastern China, due to the high population, the averted mortality rate in this region is smaller than the other countries, due to the more moderate impact of fire on air quality (Section 3.3). The greatest impact per capita is in Laos and Myanmar where 25 (95UI: 21–29) and 26 (95UI: 23–29) premature deaths per 100,000 head of population are averted per year, respectively. In Cambodia, Thailand, Vietnam and south‐eastern China, the averted mortality rate ranges from 10 to 17 (95UI: 9–18) premature deaths per 100,000 people per year.

Eliminating fire emissions reduces the annual disease burden due to long‐term exposure to ambient O_3_ by 10% in Mainland Southeast Asia (ranging from 4% in Vietnam to 20% in Myanmar), averting a total of 2,250 (95UI: 2,000–2,470) premature deaths (Table 2). In south‐eastern China, the annual disease burden is reduced by 1%, averting 1,530 (95UI: 1,380–1,660) premature deaths. In the FINNx1.5 scenario, the reduction in surface O_3_ by country is slightly smaller than for the FINN scenario due to nonlinear effects driving O_3_ concentrations, resulting in smaller averted disease burdens (Table S2).

### 3.5 Poverty and Smoke Exposure

**Figure 8:**
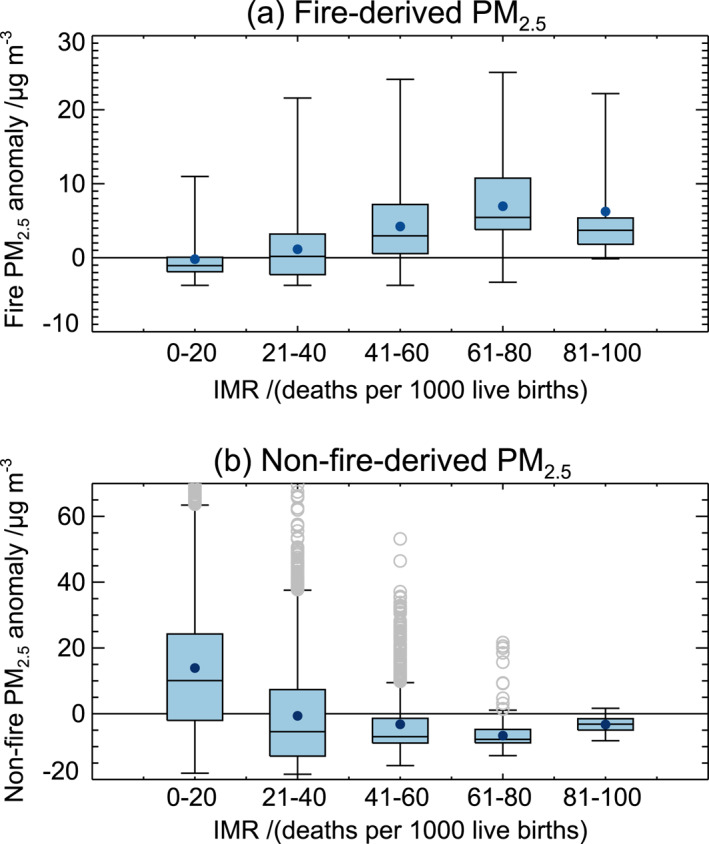
Weather Research and Forecasting model coupled with Chemistry (WRF‐Chem)‐simulated annual mean (a) fire‐derived PM_2.5_ and (b) nonfire‐derived PM_2.5_ concentrations versus binned subnational infant mortality rate (IMR) values across the Southeast Asian domain. Shown are the simulated PM_2.5_ anomalies, that is, the difference of the PM_2.5_ concentration in each IMR bin from the mean PM_2.5_ concentration across all IMR bins. Boxes enclose the interquartile range; filled circles show the mean; error bars extend to 1.5 times the 25th and 75th percentiles; gray open circles show outliers. Prior to analysis IMR values were regridded to the WRF‐Chem grid by taking the mean gridded IMR value per 0.25° × 0.25° grid cell.

In this section, we examine the poverty levels of the Southeast Asian population exposed to fire‐derived PM_2.5_ pollution. Figure 8 shows WRF‐Chem simulated annual mean fire‐derived (smoke) PM_2.5_ and nonfire PM_2.5_ concentrations plotted against gridded poverty proxy (IMR) data for the Southeast Asian domain. Populations in regions with relatively high IMRs (>60 deaths per 1,000 births) are generally exposed to higher annual mean PM_2.5_ concentrations from fire than populations with relatively low IMRs (<40 deaths per 1,000 births). In areas with IMR ≥ 60, the mean fire‐derived PM_2.5_ exposure (10.6 μg m^−3^) is significantly greater (at the 99% confidence level) than the mean fire‐derived PM_2.5_ exposure in areas with IMR ≤ 20 (3.5 μg m^−3^). At the national scale, countries with higher IMRs (Laos, Cambodia, and Myanmar; Figure S8) also experience greater particulate emissions from fires (Figure 1b) and greater exposure to fire‐derived PM_2.5_ (Figure 6a) than other countries in Southeast Asia. Also, this result may reflect that rural populations in Southeast Asia, which are generally located closer to forest and vegetation fires, often experience greater IMRs (e.g., Myanmar Ministry of Health, 2003).

When we consider PM_2.5_ from all sources other than fires (Figure 8b), we obtain the opposite result, where populations in regions with relatively high IMRs (>60 deaths per 1,000 births) are generally exposed to lower annual mean nonfire PM_2.5_ concentrations than populations with relatively low IMRs (<40 deaths per 1,000 births). In areas with IMR ≥ 60, the mean nonfire PM_2.5_ exposure (15.1 μg m^−3^) is significantly lower (at the 99% confidence level) than the mean nonfire PM_2.5_ exposure in areas with IMR ≤ 20 (35.3 μg m^−3^).

Considering PM_2.5_ from all sources (Figure S9), we find that on average, “not poor” and “moderately poor” populations (with IMR < 32) are exposed to annual mean PM_2.5_ concentrations derived predominantly (88%) from nonfire sources. However, for “very poor” populations (with 65 ≤ IMR < 100), fire‐derived PM_2.5_ makes up a more substantial fraction (41%) of the total PM_2.5_ exposure, with 59% from nonfire sources.

**Figure 9:**
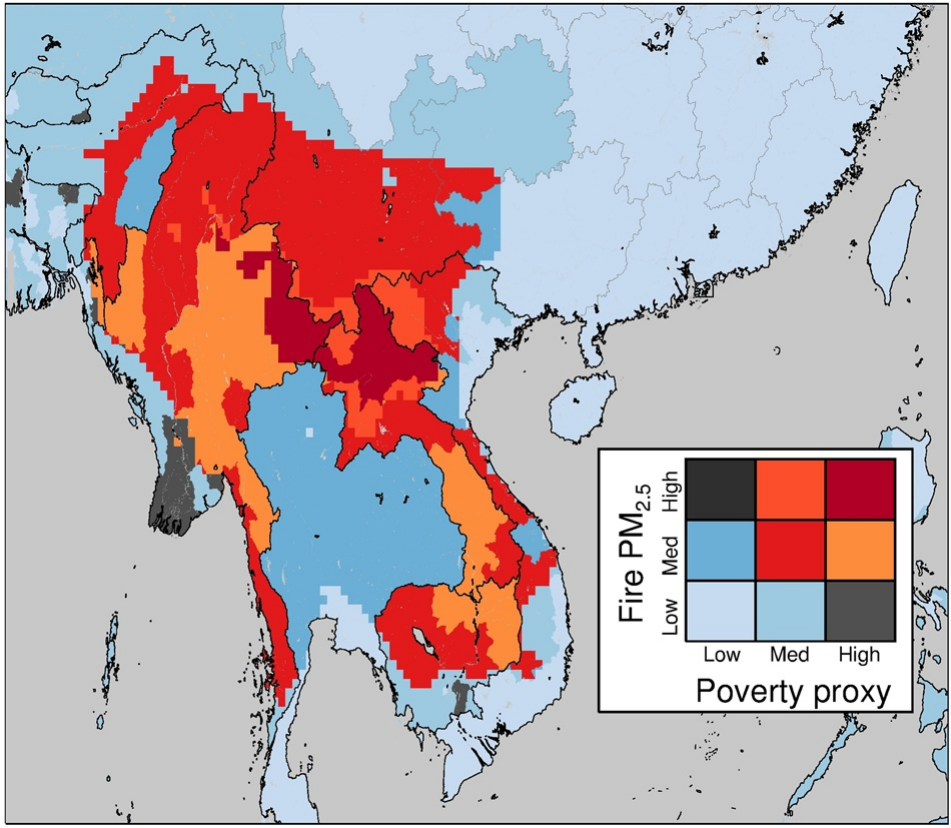
Spatial distribution of poverty proxy data (infant mortality rate [IMR] estimates) and Weather Research and Forecasting model coupled with Chemistry‐simulated annual mean fire‐derived PM_2.5_ concentrations across Southeast Asia. Poverty proxy (IMR) ranges are: low: 0–20; med = 20–60; high = 60–100 deaths per 1,000 live births. PM_2.5_ concentration ranges are: low = 0–5 μg m^−3^; med = 5–15 μg m^−3^; high = 15–30 μg m^−3^.

Figure 9 shows the spatial distribution of relative poverty levels (IMR) and fire‐derived PM_2.5_ exposure (WRF‐Chem‐simulated annual mean fire‐derived PM_2.5_ concentrations) across Southeast Asia. This figure indicates a large region in Southeast Asia (including northern Laos, north‐west Vietnam, northern Cambodia, northern and eastern Myanmar, and Yunnan province in China) where populations with medium or high levels of poverty are exposed to medium or high levels of PM_2.5_ pollution from fires. In particular, two areas in northern Laos and western Myanmar show relatively high levels of both poverty and PM_2.5_ exposure, suggesting populations in these regions may be particularly at risk to health impacts from fires.

Overall, these results suggest that populations with greater levels of poverty are disproportionally exposed to PM_2.5_ from vegetation and forest fires in Southeast Asia. For very poor populations, fire‐derived PM_2.5_ concentrations contribute over a third to the total PM_2.5_ exposure.

## 4 Discussion of Public Health Impacts and Policy

To put our estimated public health impacts into context, we compare disease burdens due to fire‐derived PM_2.5_ exposure calculated for other fire‐intensive regions. Previous studies have estimated that preventing forest and vegetation fires would avert ∼5,000–16,800 annual premature deaths across South America (Butt et al., 2020; F. H. Johnston et al., 2012; Nawaz & Henze, 2020; Reddington et al., 2015) and ∼6,000–100,300 annual premature deaths across Equatorial Asia (Crippa et al., 2016; Kiely et al., 2020; Koplitz et al., 2016; Marlier et al., 2012). The wide range in estimates reflects differences in the experimental design/methods, for example, time periods (with strong interannual variability in fire emissions in these regions), atmospheric models, and, in particular, exposure‐outcome associations (as discussed by Butt et al., 2020; Conibear et al., 2018; Giani et al., 2020; Kiely et al., 2020; Reddington, Conibear, et al., 2019).

Using similar WRF‐Chem setups and exposure‐outcome association (the GEMM) as used in this study, previous studies found that eliminating fire would avert 16,800 (95UI: 16,300–17,400) premature deaths across South America in 2012 (Butt et al., 2020) and 44,000 (34,700–53,900) premature deaths across Equatorial Asia in 2015 (Kiely et al., 2020). The total averted disease burden for our Southeast Asian domain, 59,000 (95UI: 55,200–62,900) premature deaths, is greater than estimated for the other two fire‐influenced regions, despite there being a major drought‐induced haze event across Equatorial Asia in 2015. Removing the population size dependence, the per capita averted disease burden estimates for countries in Southeast Asia (10–26 (95UI: 9–29) deaths per 100,000 people) are comparable to those estimated for Bolivia, Brazil and Peru (11–22 (95UI: 10–26) deaths per 100,000 people) in 2012 (Butt et al., 2020) and for Singapore, Brunei and Malaysia (20–33 (95UI: 16–41) deaths per 100,000 people) in 2015 (Kiely et al., 2020). These comparisons indicate that populations in Mainland Southeast Asia, suffer from substantial exposure to smoke from fires with adverse impacts on public health that are comparable to other major fire regions in the tropics.

We also compared the averted disease burden from eliminating fire to those that would be achieved by eliminating other emissions sectors, estimated in Reddington, Conibear, et al. (2019). Using the same health impact calculation method as Reddington, Conibear, et al. (2019) (the Integrated Exposure‐Response function [GBD 2015 Risk Factors Collaborators, 2016]), the avoided PM_2.5_ disease burdens in Mainland Southeast Asia due to eliminating fire emissions (12,200 [95UI: 6,500–19,000] premature deaths) are lower than calculated with the GEMM (Table 2). These values are comparable to eliminating all industrial emissions; a factor 6 greater than eliminating electricity generation emissions; and a factor 10 greater than eliminating land transport across Mainland Southeast Asia. We note that we do not account for toxicity variation within PM_2.5_ exposure as it is currently unknown; with disagreement in the literature regarding the toxicity of fire‐derived PM relative to ambient PM (Aguilera et al., 2021; H. J. Johnston et al., 2019; Pongpiachan, 2016; Wegesser et al., 2009). The health effects of different sources and components of PM exposure is an ongoing area of research (Adetona et al., 2016; J. C. Liu et al., 2015; Naeher et al., 2007; Reid et al., 2016).

There is considerable uncertainty associated with deriving fire emissions from satellite retrievals (e.g., Pan et al., 2020; Reddington et al., 2016), and previous studies have reported that these emissions, particularly from agricultural fires, may be underestimated in Mainland Southeast Asia (Lasko et al., 2017; Reddington et al., 2016; Sornpoon et al., 2014) and China (Shen et al., 2019; Stavrakou et al., 2016; L. Zhang et al., 2016; T. Zhang et al., 2020). The underestimation of emissions from these fires is likely due to multiple factors, but particularly their small size (difficult for burned area products to detect) and short duration of active burning (a high potential to be missed by polar‐orbiting satellites with detection frequencies of only a few times per day) (e.g., T. Zhang, Wooster, et al., 2018). Applying a simple scaling factor to the fire emissions will partly compensate for emissions underestimation, but emissions estimates are still likely to be conservative in regions with a high number of missed detections.

Our analysis shows that a reduction of fire across southeast Asia would have substantial health benefits. Successful fire management requires information about the main types and causes of fire. Across Mainland Southeast Asia, emissions are dominated by forest fires (deforestation, savanna, and temperate forest classes in GFED) which account for 96% of particulate emissions across our domain, with greater contributions in Cambodia, Laos, and Myanmar. A detailed analysis of fires confirms that most fires in the region occur in forest land covers (Vadrevu et al., 2019). A close association between fire and deforestation has also been shown in other tropical regions including the Brazilian Amazon (Reddington et al., 2015) and Indonesia (Adrianto et al., 2019, 2020). In Southeast Asia, fires are lit in forests to clear the land for agriculture (slash and burn, deforestation fires), to induce growth of grass for grazing, and for collection of forest products (Vadrevu et al., 2019). The large contribution of forest fires to particulate emissions suggests that reducing deforestation and associated fires should be a public health priority for the region. In Cambodia, deforestation has been linked to increased incidence of acute respiratory infection in children, likely due to increased exposure to smoke from deforestation fires (Pienkowski et al., 2017). Future work exploring the relative contributions of different fire types to air pollution in Mainland Southeast Asia would be useful to inform policy options to improve air quality.

Several policies have already been implemented to reduce agricultural fires in Southeast Asia, for example, an Alternative Energy Development Plan and a zero‐burning policy for sugarcane in Thailand (Kumar et al., 2020). However, challenges remain with regards to the enforcement of these policies and their practicality, particularly for farmers that rely on manual harvesting practices (Adeleke et al., 2017; Kumar et al., 2020). Recent research shows the most effective solutions for reducing agricultural residue burning and its associated air pollution, are to encourage residue use for other purposes, for example, bioenergy, livestock feed/bedding, composting, green harvesting and so on (Kumar et al., 2020) and to apply coherent policies across multiple provinces and countries in Southeast Asia (Moran et al., 2019).

Discussion of the implementation and benefits of policies addressing deforestation and/or savanna‐type fires in Southeast Asia are lacking in the literature. However, a number of policies and projects have been developed and implemented to address forest loss and conversion, many of which are related to UNFCCC REDD+ (reducing emissions from deforestation and forest degradation and the role of conservation, sustainable management of forest and enhancement of forest carbon stocks) (e.g., Kissinger, 2020). Key drivers of deforestation are expansion of cropland and commercial agriculture (Lim et al., 2017; Y. Zhang, Prescott, et al., 2018), for example, conversion of forest to coffee and/or rubber plantations (Fox & Castella, 2013; Kissinger, 2020). There is evidence that protected areas and community‐protected forests can play an important role in protecting forests from large‐scale burning and deforestation fires (Biswas et al., 2015; Singh et al., 2018).

## 5 Conclusions

In this study, we explored the impact of forest and vegetation fires on air quality and public health across Southeast Asia. We used a combination of two air quality models: a global aerosol model, GLOMAP, to test three different satellite‐derived fire emission data sets (FINN, GFED, GFAS); and a high‐resolution, regional air quality model, WRF‐Chem, to quantify the air quality and public health benefits of eliminating fire emissions. Simulating the elimination of all fires across the region, rather than fires specifically identified to be human‐caused, illustrates the maximum possible public health benefit achievable (within uncertainties) and provides an upper bound for policy makers.

We found that GLOMAP was better able to reproduce measurements of fire‐derived PM in Thailand across multiple years with the FINN data set compared to the GFAS or GFED data sets. This result is consistent with findings in our previous work (Reddington et al., 2016). PM emissions across Southeast Asia in FINN are a factor 4 greater than GFED or GFAS. WRF‐Chem using FINN best simulated measured PM concentrations when particulate fire emissions were scaled upwards by a factor 1.5. Our analysis suggests fire emissions in this region are underestimated, particularly in the GFED and GFAS data sets.

Overall, we found that preventing fire could substantially improve regional air quality in Mainland Southeast Asia with a more limited benefit to air quality in south‐eastern China. Population‐weighted annual mean PM_2.5_ concentrations were reduced by 16% in Mainland Southeast Asia and by 2% in south‐eastern China. ADM8h O_3_ concentrations were reduced by 9% in Mainland Southeast Asia and by 2% in south‐eastern China. Eliminating fire emissions substantially reduced populations exposed to PM_2.5_ concentrations above WHO AQ Interim Target 2 in Thailand, Myanmar, Laos, and Cambodia (by 44%–100%).

We found a considerable public health benefit of eliminating fire emissions across the region, largely due to reductions in PM_2.5_ exposures. The annual disease burden due to PM_2.5_ exposure was reduced by 12% in Mainland Southeast Asia, averting 27,500 (95UI: 24,700–30,400) premature deaths, and by 3% in south‐eastern China, averting 31,400 (95UI: 30,500–32,400) premature deaths. The annual disease burden due to O_3_ exposure was reduced by 10% in Mainland Southeast Asia, averting 2,250 (95UI: 2,000–2,470) premature deaths, and by 1% in south‐eastern China, averting 1,530 (95UI: 1,380–1,660) premature deaths.

Using subnational poverty proxy data, we found that poorer populations in Southeast Asia are disproportionally exposed to PM_2.5_ from vegetation and forest fires; with significantly higher average fire‐derived PM_2.5_ exposure for populations with relatively high infant mortality rates.

Our analysis suggests that exposure to fire‐derived PM_2.5_ is associated with a greater annual disease burden in Southeast Asia than in both the Amazon region in 2012 and Equatorial Asia in 2015, with similar per capita averted disease burdens to those estimated for heavily fire‐impacted countries in South America. Furthermore, preventing fires across Mainland Southeast Asia would yield a public health benefit comparable to that achieved by eliminating all industrial emissions across the region, and considerably larger than achieved by eliminating emissions from either the electricity generation or land transport sectors.

In summary, forest and vegetation fires are important to consider in addition to more traditional emission sectors (e.g., industry, transport, and residential solid‐fuel combustion) when assessing causes of air quality degradation in Southeast Asia and for developing emission control policies to improve air quality across this region. These policies should focus on reducing deforestation and savanna‐type fires in addition to agricultural fires in order to effectively address the regional air quality issues. Previous work in Equatorial Asia (Reddington et al., 2014) demonstrates the need to understand the effectiveness of regional emission control strategies and how they will reduce population exposure. Future work is required to identify the regions where emission controls would most effectively reduce exposure, especially for the poorest populations.

## Conflict of Interest

The authors declare no conflicts of interest relevant to this study.

## Supporting information

Supporting Information S1

## Data Availability

The air pollution and health impact assessment data per country/region that support the findings of this study are available at the Research Data Leeds Repository (https://doi.org/10.5518/968). Code to setup and run WRFChem (using WRFotron version 2.0) is available through Conibear and Knote (2020).
