# Supplementary material for: Air Pollution From Forest and Vegetation Fires in Southeast Asia Disproportionately Impacts the Poor
Source: Geohealth. 2021 Sep 1;5(9):e2021GH000418. doi: 10.1029/2021GH000418 (PMC8411873; doi:10.1029/2021GH000418)
Supplement: Supplementary file 1 — Supporting Information S1 [file GH2-5-e2021GH000418-s001.pdf]

GeoHealth

Supporting Information for

Air Pollution from Forest and Vegetation Fires in Southeast Asia Disproportionately Impacts the Poor

Carly L. Reddington*[,1], Luke Conibear[1], Suzanne Robinson[1], Christoph Knote[2], Stephen R. Arnold[1], and Dominick V. Spracklen[1]

[1] Institute for Climate and Atmospheric Science, School of Earth and Environment, University of Leeds, Leeds, UK

[2] Faculty of Medicine, University of Augsburg, Germany

* Corresponding author: Carly Reddington (C.L.S.Reddington@leeds.ac.uk)

Contents of this file	Page No.
**S1. Methods**	2-5
**S1.1 Description of the GLOMAP global aerosol model**	2
**S1.2 Description of the WRF-Chem regional model**	2
**S1.3 Calculation of public health impacts**	3
**S1.4 Measurements of particulate matter and ozone concentrations**	4
**S1.5 Global Subnational Infant Mortality Rates**	5
**S2. Extended model evaluation**	5-6
**S2.1 Extended evaluation of GLOMAP fire-derived PM_{10}**	5
**S2.2 Evaluation of WRF-Chem $PM_{2.5}$**	6
**Supporting Tables**	7-8
**Table S1.** Summary of annual mean $PM_{2.5}$ measurements from the World Health Organization (WHO) Ambient Air Quality Database (WHO, 2018).	7
**Table S2.** Averted public health effects due to changes in long-term exposure to ambient $PM_{2.5}$ and ozone from eliminating fire emissions.	8
**Supporting Figures**	9-17
**Figure S1.** Locations of the air quality monitoring stations used to evaluate the models.	9
**Figure S2.** Taylor diagram comparing GLOMAP-simulated and measured multi-annual average seasonal cycles of fire-derived PM_{10} concentrations at 12 air quality monitoring stations in northern Thailand.	10
**Figure S3.** WRF-Chem-simulated and measured monthly mean *total* PM_{10} concentrations during 2014 averaged over 12 air quality monitoring stations in fire-influenced regions of Thailand.	10
**Figure S4.** Taylor diagram comparing WRF-Chem-simulated and measured monthly mean fire-derived PM_{10} concentrations during 2014 at 12 air quality monitoring stations in fire-influenced regions of Thailand.	11

**Figure S5.** WRF-Chem-simulated and measured annual mean surface $PM_{2.5}$ concentrations across Southeast Asia.	12
**Figure S6.** WRF-Chem-simulated and measured daily mean surface ozone concentrations during April to July 2014 over south-eastern China.	13
**Figure S7.** Spatial distribution of WRF-Chem-simulated annual mean surface $PM_{2.5}$ and ozone concentrations across Southeast Asia for 2014.	15
**Figure S8.** Spatial distribution of subnational infant mortality rate estimates across Southeast Asia for the year of 2015.	16
**Figure S9.** Gridded subnational infant mortality rate values versus WRF-Chem simulated annual mean $PM_{2.5}$.	17
**References**	18

S1. Methods

S1.1 Description of the GLOMAP global aerosol model

The Global Model of Aerosol Processes (GLOMAP) (Spracklen et al., 2005; Mann et al., 2010) is an extension of the TOMCAT global 3-D offline chemical transport model (Chipperfield, 2006), resolving aerosol chemistry and microphysics. The GLOMAP aerosol model has a horizontal resolution of 2.8°×2.8° with 31 vertical model levels between the surface and 10 hPa. Large-scale atmospheric transport and meteorology in are specified from European Centre for Medium-Range Weather Forecasting (ECMWF) ERA-Interim global reanalysis data (Dee et al., 2011), updated every six hours and linearly interpolated onto the model time step. The aerosol size distribution is represented by a two-moment modal aerosol scheme (Mann et al., 2010). GLOMAP includes black carbon (BC), primary and secondary organic aerosol, sulfate (SO_4), sea spray and mineral dust. Concentrations of oxidants are specified using monthly mean 3-D fields at 6-hourly intervals from a TOMCAT simulation with detailed tropospheric chemistry (Arnold et al., 2005) linearly interpolated onto the model time step.

Anthropogenic emissions of sulfur dioxide (SO_2), BC and organic carbon (OC) were specified using annually varying MACCity emissions inventory for the years 2002-2010 (Granier et al., 2011). For simulations in 2011 and beyond, we used MACCity anthropogenic emissions from 2010. Monthly mean emissions of biogenic monoterpenes are taken from the Global Emissions InitiAtive (GEIA) database (Guenther et al., 1995). Monoterpenes are oxidised to form a product that condenses irreversibly in the particle phase to form secondary organic aerosol (Scott et al., 2014). Size-resolved emissions of mineral dust are prescribed from daily varying emissions fluxes provided for AEROCOM (Dentener et al., 2006).

S1.2 Description of the WRF-Chem regional model

In the version of WRF-Chem used in this study, aerosol physics and chemistry are treated using the Model for Simulating Aerosol Interactions and Chemistry (MOSAIC; Zaveri et al., 2008) scheme, using chemistry option 201, with an extended treatment of organic aerosol (Hodzic and Jimenez, 2011; Hodzic and Knote, 2014). The MOSAIC scheme treats major aerosol species including SO_4, nitrate, chloride, ammonium, sodium, BC, primary and secondary organic aerosol (formed from biogenic, anthropogenic and biomass burning

precursors), and other inorganics (including crustal and dust particles and residual primary $PM_{2.5}$). Four discrete size bins are used within MOSAIC to represent the aerosol size distribution (with the following dry particle diameter ranges: 0.039–0.156 μm, 0.156–0.625 μm, 0.625–2.5 μm, and 2.5–10 μm). Gas-phase chemical reactions are calculated using the extended Model for Ozone and Related Chemical Tracers (MOZART) (Emmons et al., 2010) chemical mechanism, with several updates to photochemistry of aromatics, biogenic hydrocarbons and other species relevant to regional air quality (Hodzic and Jimenez, 2011; Knote et al., 2014).

Simulated mesoscale meteorology is kept in line with analysed meteorology through grid nudging to the National Centre for Environmental Prediction (NCEP) Global Forecast System (GFS) analyses to limit errors in mesoscale transport (NCEP, 2000; 2007). The model meteorology was reinitialised every month to avoid drifting of WRF-Chem and spun up for 12 hours, while chemistry and aerosol fields were retained to allow for pollution build-up and mesoscale pollutant transport phenomena to be captured. MOZART-4/Goddard Earth Observing System Model version 5 (GEOS5) 6-hourly simulation data (NCAR, 2016) were used for chemical and aerosol boundary conditions.

Anthropogenic emissions were taken from the Emission Database for Global Atmospheric Research with Task Force on Hemispheric Transport of Air Pollution (EDGAR-HTAP) version 2.2 at 0.1°×0.1° horizontal resolution (Janssens-Maenhout et al., 2015). Biogenic emissions were calculated online by the Model of Emissions of Gases and Aerosol from Nature (MEGAN; Guenther et al., 2006). Dust emissions were calculated online through the Georgia Institute of Technology-Goddard Global Ozone Chemistry Aerosol Radiation and Transport (GOCART) model with Air Force Weather Agency (AFWA) modifications (LeGrand et al., 2019).

S1.3 Calculation of public health impacts

The relative risk of disease at a specific ambient $PM_{2.5}$ exposure was estimated through the Global Exposure Mortality Model (GEMM) (Burnett et al., 2018). The population attributable fraction (PAF) was estimated per grid cell as a function of population (P) and the relative risk (RR) of exposure following Equation 1. We used the GEMM for non–accidental mortality (non–communicable disease, NCD, plus lower respiratory infections, LRI), using parameters including the China cohort (Yin et al., 2017), with age–specific modifiers for adults over 25 years of age in 5–year intervals. The GEMM functions have mean, lower, and upper uncertainty intervals. The minimum-risk exposure for the GEMM functions is 2.4 μg m^{-3}.

$$PAF = P \times (RR_{EXP} - 1/RR_{EXP}) \quad (1)$$

For ambient ozone (O_3) exposure, the PAF was estimated as a function of the summary hazard ratio (HR) for chronic obstructive pulmonary disease (COPD) only and the change in annual average, daily maximum, 8–hour, O_3 concentrations (ADM8h) relative to the minimum–risk exposure (ΔX) as shown by Equation 2. The HR for COPD was 1.14 (95UI: 1.08–1.21) (Turner et al., 2016). The minimum-risk exposure followed the minimum percentiles of 26.7 ppb.

$$PAF = P \times \left(1 - e^{\Delta X \times ln(HR)/10}\right) \quad (2)$$

Premature mortality (MORT), years of life lost (YLL), and years lived with disability (YLD) per health outcome, age bracket, and grid cell were estimated as a function of the PAF and corresponding baseline mortality (I) following Equations 3, 4, and 5, respectively. Disability–adjusted life years (DALYs), i.e., the total loss of healthy life, were estimated as the total of YLL and YLD following Equation 6. Mean estimates were quantified in addition to upper and lower uncertainty intervals at the 95% confidence level. The rates of MORT, YLL, YLD, and DALYs were calculated per 100,000 population.

$$MORT = PAF \times I_{MORT} \quad (3)$$

$$YLL = PAF \times I_{YLL} \quad (4)$$

$$YLD = PAF \times I_{YLD} \quad (5)$$

$$DALYs = YLL + YLD \quad (6)$$

The United Nations adjusted population count dataset for 2015 at 0.25° × 0.25° resolution was obtained from the Gridded Population of the World, Version 4 (GPWv4) (Center for International Earth Science Information Network (CIESIN), 2016a). Population age composition for 2015 for adults 25 to 80 years in 5–year intervals, and 80 years plus, was taken from the Global Burden of Disease (GBD) Study 2017 (GBD 2017 Risk Factors Collaborators, 2018). Cause–specific (NCD, LRI, and COPD) baseline mortality and morbidity rates for 2015 for MORT, YLL, and YLD for each age bracket were also taken from the GBD Study 2017 (Institute for Health Metrics and Evaluation, 2019). Shapefiles were used to aggregate results at the country and state level (Hijmans et al., 2016).

S1.4 Measurements of particulate matter and ozone concentrations

To evaluate model-simulated monthly mean surface PM_{10} concentrations (Sects. 3.2.1 and 3.2.2), we used data from the Pollution Control Department (PCD) of the Thailand Government Ministry of Natural Resources and Environment (http://www.pcd.go.th/index.cfm). The PCD air quality database (available at: http://air4thai.pcd.go.th/webV2/history/) contains historical monthly mean PM_{10} concentrations measured at ground-based air quality monitoring stations located across Thailand (see Fig. S1a). To evaluate the GLOMAP model we used measurements from stations with data available between January 2003 and December 2015 (inclusive). To evaluate the WRF-Chem model we used measurements from stations with data available between January 2014 and December 2014 (inclusive).

To evaluate WRF-Chem-simulated surface ozone concentrations (Sect. 3.2.3), we used data from the Berkley Earth China Air Quality Data Set (available at: http://berkeleyearth.lbl.gov/manual/china_air_quality/) (Rohde and Muller, 2015). This dataset consists of hourly real-time ozone data recorded at surface air quality monitoring stations located in urban areas in China and surrounding countries (see Fig. S1b). The ozone data was downloaded by Rohde and Muller (2015) from https://aqicn.org/ between 5th April and 18th July 2014. Some quality control and validation checks were applied to the raw data prior to incorporation into the Berkley Earth China Air Quality Data Set (see further details in Rohde and Muller (2015)). We calculated daily mean values from the hourly data.

To evaluate WRF-Chem-simulated surface $PM_{2.5}$ concentrations (Sect. S2.2), we used a subset of measured annual mean $PM_{2.5}$ concentrations from the World Health Organization (WHO) Global Ambient Air Quality Database (WHO, 2018). The database consists of city-average annual mean $PM_{2.5}$ concentrations obtained from multiple ground station measurements across different years. To compare with the model concentrations, we selected measurement years to match or to be as close as possible to the simulation year of 2014. For some locations, $PM_{2.5}$ concentrations have been calculated by the WHO from the measured PM_{10} concentration using national conversion factors ($PM_{2.5}/PM_{10}$ ratio) either provided by the country or estimated as population-weighted averages of urban-specific conversion factors (estimated as the mean $PM_{2.5}/PM_{10}$ ratio of stations for the same year) for the country (WHO, 2016; 2018).

Prior to all model-measurement comparisons, simulated surface PM/ozone concentrations were linearly interpolated to the location (longitude and latitude) of the individual air quality monitoring stations; averaged over the corresponding time period (daily, monthly or annual); and simulated data corresponding to time periods of missing measurement data was removed.

S1.5 Global Subnational Infant Mortality Rates

We used the Global Subnational Infant Mortality Rates (IMR), Version 2, dataset from NASA Socioeconomic Data and Applications Center (SEDAC) (CIESIN, 2018a; Fig. S8), which is benchmarked to the year 2015. We selected the year 2015 (from two years available: 2000 and 2015) to be as close as possible to the WRF-Chem model simulation year (2014) and to be consistent with the 2015 population count dataset used to calculate public health impacts (Sect. S1.3). National median estimates of IMR show little change between 2014 and 2015 (ranging from a 1% change in Vietnam to an 8% change in China) (United Nations Inter-agency Group for Child Mortality Estimation, 2020).

The dataset includes IMR data for the lowest administrative units available for each country as of June 2017 (CIESIN, 2018b) at a spatial resolution of 30 arc-seconds (~1 km). The data were drawn from national offices, Demographic and Health Surveys (DHS), Multiple Indicator Cluster Surveys (MICS), and other sources from 2006 to 2014 (CIESIN, 2018b), with boundary inputs from the GPWv4 (CIESIN, 2016a; CIESIN, 2016b).

S2. Extended model evaluation

S2.1 Extended evaluation of GLOMAP fire-derived PM_{10}

Figure S2 summarises the agreement between the average seasonal cycles in GLOMAP-simulated and measured fire-derived PM_{10} concentrations at each of the 12 fire-influenced monitoring stations. The temporal correlation at each station is similar between the model simulations with fire (GFED: r=0.90-0.97; GFAS: r=0.80-0.93; FINN: r=0.80-0.99), but the observed magnitude and variability in monthly mean PM_{10} concentrations are captured best in the simulation with FINN emissions (GFED: normalised standard deviation (NSD)=0.25-0.37; GFAS: NSD=0.29-0.42; FINN: NSD=0.73-1.06).

S2.2 Evaluation of WRF-Chem $PM_{2.5}$

We evaluate annual mean $PM_{2.5}$ concentrations simulated by WRF-Chem because the estimated public health impacts of fire-derived PM (Sect. 3.4) are calculated using this quantity. Figure S5 compares annual mean surface $PM_{2.5}$ concentrations from the FINNx1.5 simulation against $PM_{2.5}$ measurements from the WHO Global Air Quality Database. The model captures the spatial distribution of measured annual mean $PM_{2.5}$ concentrations reasonably well across the region, with greatest concentrations in southern China and north-eastern India and comparatively lower concentrations over Mainland Southeast Asia (Fig. S5a). We find that the spatial agreement between the model and measurements (Fig 5b; r=0.47) is improved with 2014-only measurements (r=0.85) or only using direct measurements of $PM_{2.5}$, removing those converted from PM_{10} (r=0.86).

Simulated annual mean $PM_{2.5}$ concentrations are unbiased when compared against all the WHO measurements available within the model domain (Fig. S5b; NMBF=0.05). Table S1 summarises the agreement between model and measurements by country. The model captures the magnitude concentrations within a factor 1.5 in Vietnam, north-east India, southern China and Thailand. The model underestimates measured annual mean $PM_{2.5}$ concentrations in Myanmar by a factor 2 (NMBF=-1.01), likely due to a combination of underestimating anthropogenic and fire emissions, underestimating or missing outflow of PM from India, and mismatching measurement and simulation years. We also note that the WHO $PM_{2.5}$ concentrations reported for Myanmar are converted from PM_{10}, which can be associated with large uncertainties.

Supporting Tables

Country	No. of stations	Year(s) of measurements	Measured/ converted $PM_{2.5}$	Model (FINN) NMBF; r	Model (FINNx1.5) NMBF; r
South-eastern China	58	2014	Measured	+0.19; 0.86	+0.20; 0.86
North-eastern India	17	2012, 2014, 2015	Measured: 3<br>Converted: 14	+0.03; 0.26	+0.07; 0.28
Myanmar	16	2009, 2012, 2013, 2015	Converted	-1.23; 0.35	-1.01; 0.36
Thailand	22	2014	Converted	+0.09; 0.41	+0.16; 0.40
Vietnam	2	2016	Measured	+0.47; -	+0.50; -

Table S1. Summary of annual mean $PM_{2.5}$ measurements from the World Health Organization (WHO) Ambient Air Quality Database (WHO, 2018). The table shows the number of stations with available data, the year(s) the measurements were conducted and the number of reported $PM_{2.5}$ concentrations that were converted from PM_{10} measurements. The WRF-Chem normalised mean bias factor (NMBF; Yu et al., 2006) and Pearson's correlation coefficient (r) against observations are given for each country with available WHO measurements.

Country/ region	Reduction in $PM_{2.5}$ MORT	$PM_{2.5}$ MORT (yr^{-1})	$PM_{2.5}$ DALYs (yr^{-1})	Reduction in O_3 MORT	O_3 MORT (yr^{-1})
Cambodia	10%	1,100 (1,000-1,300)	44,500 (36,700-53,600)	15%	150 (130-160)
Laos	22%	1,200 (1,000-1,400)	47,600 (37,000-57,900)	17%	80 (70-90)
Myanmar	17%	8,000 (7,100-9,000)	293,800 (243,800-349,200)	21%	1,090 (960-1,210)
Thailand	12%	6,500 (6,000-7,000)	264,200 (221,300-311,100)	8%	620 (570-670)
Vietnam	3%	3,600 (3,300-4,100)	131,900 (108,800-159,100)	5%	410 (360-450)
Total Mainland SE Asia	9%	20,500 (18,400-22,700)	782,000 (647,700-931,000)	10%	2,350 (2,090-2,570)
SE China	3%	24,000 (23,400-24,800)	798,100 (703,500-906,800)	2%	2,170 (1,950-2,350)

Table S2. Averted public health effects due to changes in long-term exposure to ambient $PM_{2.5}$ and ozone (O_3) from eliminating fire emissions. Shown are the percentage reductions in annual disease burden, and the numbers of averted annual premature mortalities (MORT) and disability-adjusted life years (DALYs) per country for the lower fire emissions scenario (FINN). Values in parentheses represent the 95% uncertainty intervals (95UI). $PM_{2.5}$ mortality values are rounded to the nearest 100 and O_3 mortality values are rounded to the nearest 10. "SE China" is defined as south of 30°N and east of 98°W, and includes Hong Kong SAR, Macau SAR and Taiwan. "Mainland SE Asia" includes Cambodia, Laos, Myanmar, Thailand, and Vietnam.

Supporting Figures

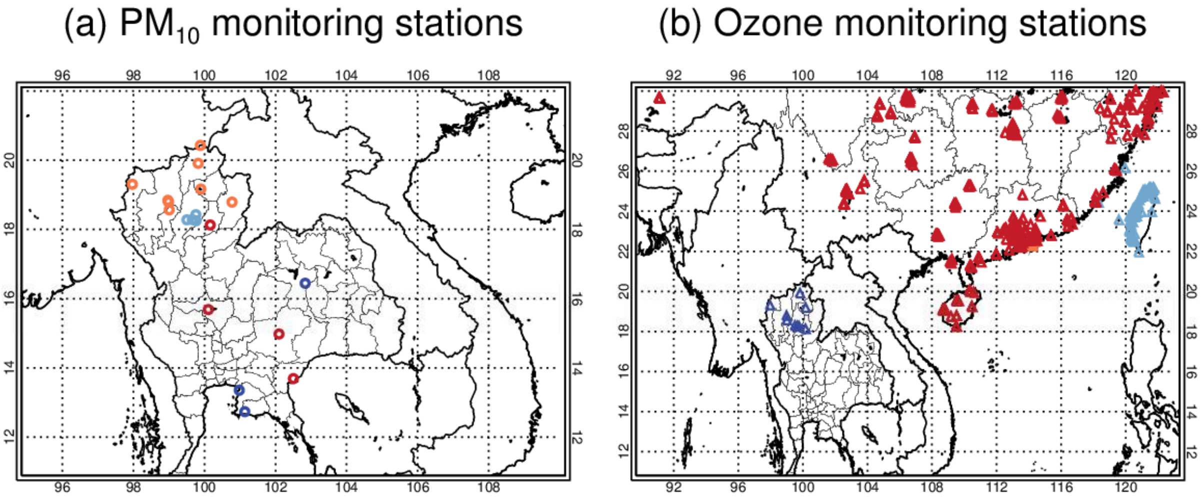

Figure S1. Locations of the air quality monitoring stations used to evaluate the models (Sects. 3.2 and 3.3). **(a)** Thailand PCD PM10 monitoring stations. Stations defined as influenced by fire emissions (where FINN fire-derived PM10 contribute ≥ 20% to the annual mean PM10) by both the GLOMAP and WRF-Chem models are coloured orange; Stations defined as fire-influenced by the WRF-Chem model only are coloured red; stations defined as fire-influenced by the GLOMAP model only are coloured light blue; the remaining stations are coloured dark blue. **(b)** Ozone monitoring stations from Rohde and Muller (2015) coloured by region: Thailand (dark blue); Mainland China (red); Hong Kong (orange); and Taiwan (light blue).

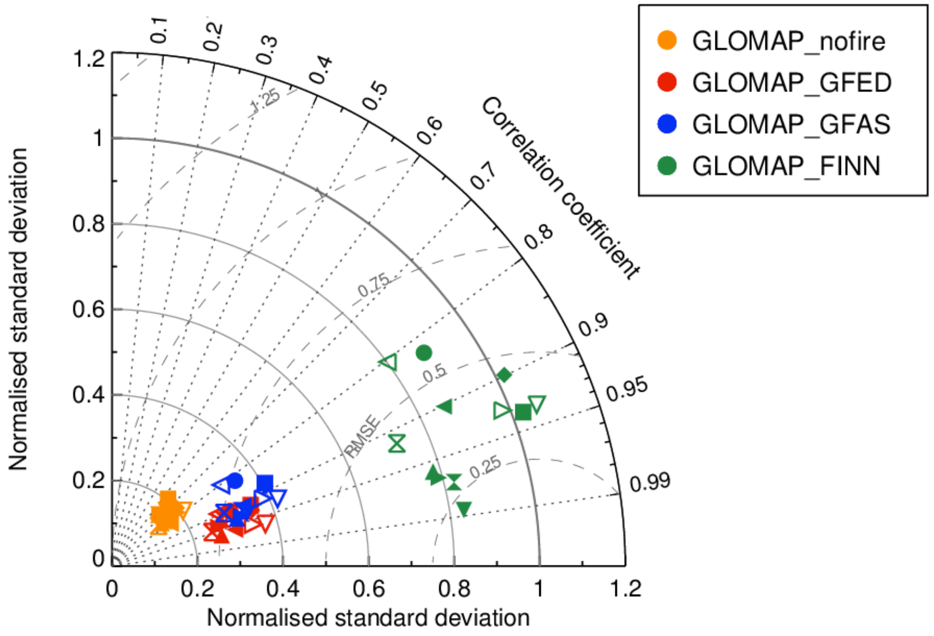

Figure S2. Taylor diagram comparing GLOMAP-simulated and measured multi-annual average seasonal cycles of fire-derived PM_{10} concentrations at 12 air quality monitoring stations in northern Thailand (Fig. S1). The measurements are represented by a point on the x-axis at unit distance from the y-axis. Results are shown for three model simulations: without fire emissions (GLOMAP_nofire); with GFED4 emissions (GLOMAP_GFED); with GFASv1.2 emissions (GLOMAP_GFAS); and with FINNv1.5 emissions (GLOMAP_FINN). The model standard deviation and centred root mean square error (RMSE) are normalised by dividing by the corresponding measured standard deviation. The normalised standard deviation and RMSE values are marked by the solid and dashed lines, respectively.

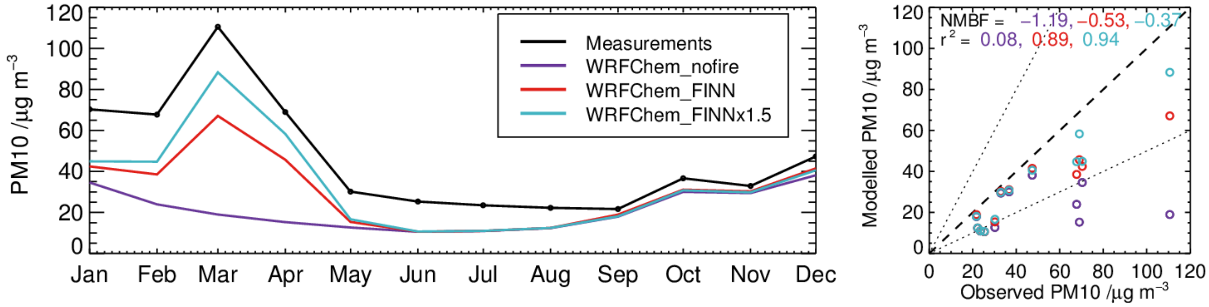

Figure S3. WRF-Chem-simulated and measured monthly mean *total* PM_{10} concentrations during 2014 averaged over 12 air quality monitoring stations in fire-influenced regions of Thailand (Fig. S1). Simulated concentrations are shown for the model without fire emissions (WRFChem_nofire), and for the model with FINN fire emissions (WRFChem_FINN) and with FINN emissions scaled upwards by a factor 1.5 (WRFChem_FINNx1.5).

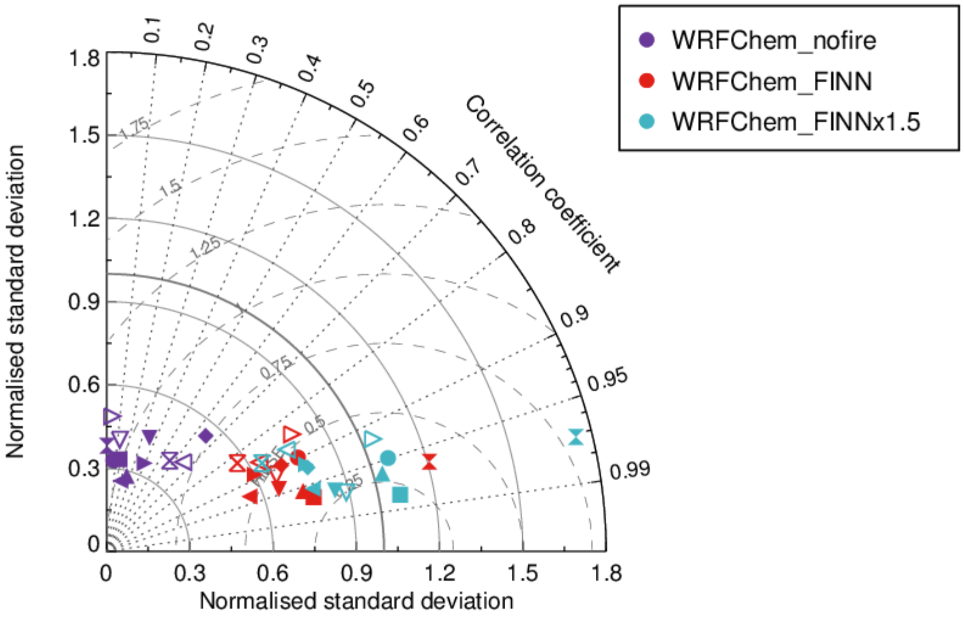

Figure S4. Taylor diagram comparing WRF-Chem-simulated and measured monthly mean fire-derived PM_{10} concentrations during 2014 at 12 air quality monitoring stations in fire-influenced regions of Thailand (Fig. S1). The measurements are represented by a point on the x-axis at unit distance from the y-axis. Results are shown for three model simulations: without fire emissions (WRFChem_nofire); with FINN fire emissions (WRFChem_FINN); and with FINN emissions scaled upwards by a factor 1.5 (WRFChem_FINNx1.5). The model standard deviation and centred root mean square error (RMSE) are normalised by dividing by the corresponding measured standard deviation. The normalised standard deviation and RMSE values are marked by the solid and dashed lines, respectively.

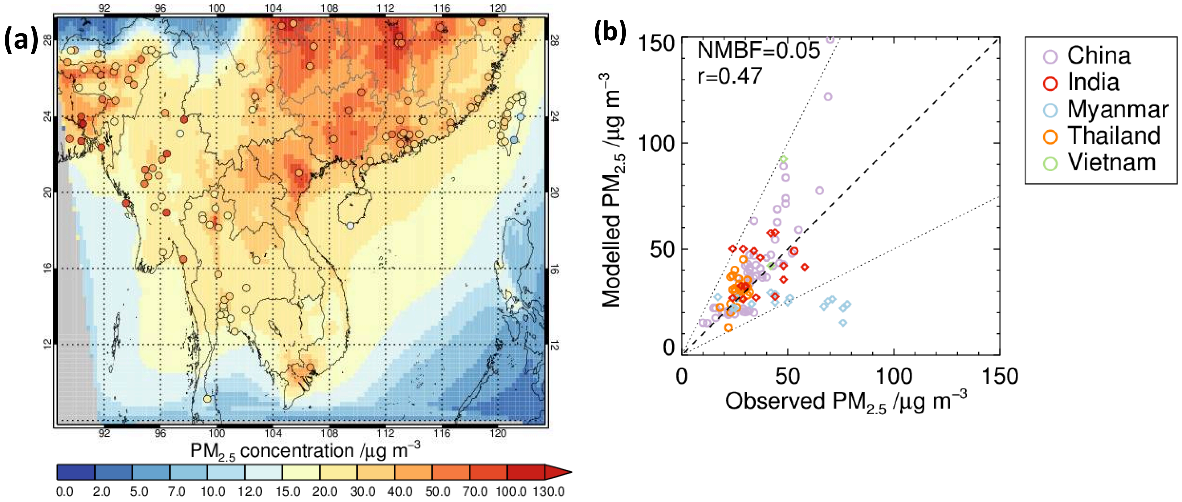

Figure S5. WRF-Chem-simulated and measured annual mean surface $PM_{2.5}$ concentrations across Southeast Asia. **(a)** Map of the simulated surface distribution of annual mean $PM_{2.5}$ for 2014 (underlying colours); overlying circles show measured annual mean $PM_{2.5}$ concentrations for available years (2009-2016). Regions in grey are outside the model domain. **(b)** Simulated versus measured annual mean $PM_{2.5}$ concentrations. Circles show measured annual mean $PM_{2.5}$ concentrations for the year 2014; diamonds show measured concentrations for years other than 2014. All simulated annual mean $PM_{2.5}$ concentrations are for the year 2014. The normalised mean bias factor (NMBF) and Pearson's correlation coefficient (r) between simulated and measured values are displayed in the top left corner.

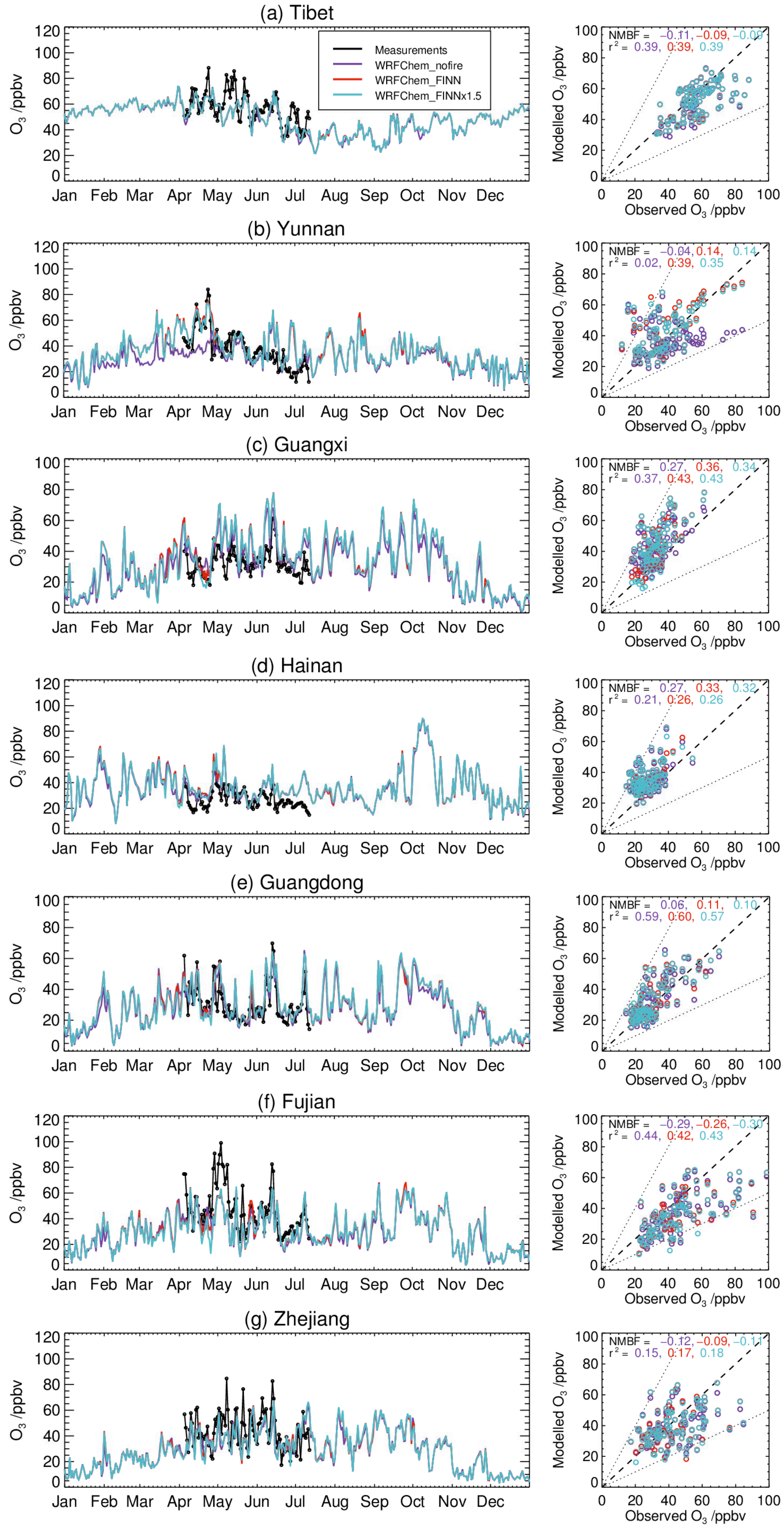

(a) Tibet
Measurements
WRFChem_nofire
WRFChem_FINN
WRFChem_FINNx1.5
NMBF = −0.11, −0.09, −0.09
r² = 0.39, 0.39, 0.39
(b) Yunnan
NMBF = −0.04, 0.14, 0.14
r² = 0.02, 0.39, 0.35
(c) Guangxi
NMBF = 0.27, 0.36, 0.34
r² = 0.37, 0.43, 0.43
(d) Hainan
NMBF = 0.27, 0.33, 0.32
r² = 0.21, 0.26, 0.26
(e) Guangdong
NMBF = 0.06, 0.11, 0.10
r² = 0.59, 0.60, 0.57
(f) Fujian
NMBF = −0.29, −0.26, −0.30
r² = 0.44, 0.42, 0.43
(g) Zhejiang
NMBF = −0.12, −0.09, −0.11
r² = 0.15, 0.17, 0.18
O3 /ppbv
Modelled O3 /ppbv
Observed O3 /ppbv
Jan Feb Mar Apr May Jun Jul Aug Sep Oct Nov Dec

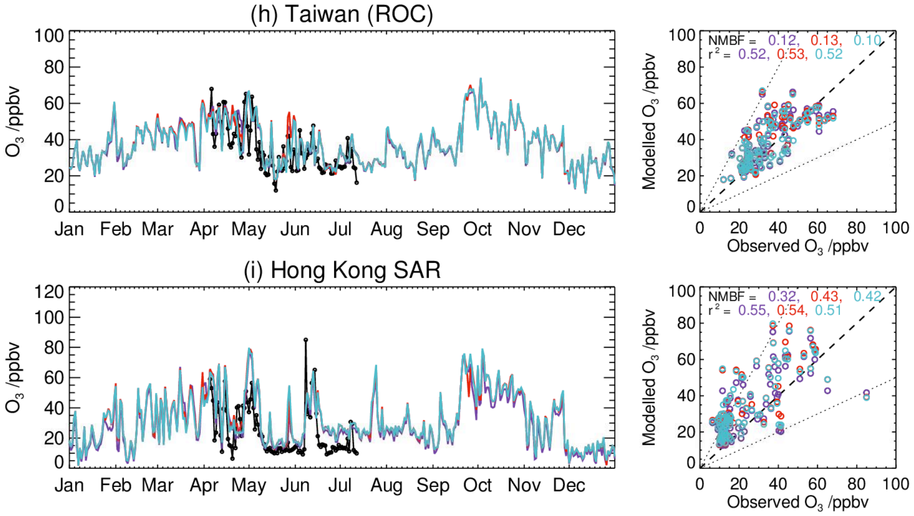

Figure S6. Evaluation of WRF-Chem-simulated ozone (O_3) over Thailand and South-eastern (SE) China. Left: Time-series of simulated and measured daily mean surface O_3 mixing ratios during 2014; Right: simulated versus measured daily mean O_3. Regional/province averages are shown for: **(a)** Tibet (7 air quality monitoring stations); **(b)** Yunnan (15 stations); **(c)** Guangxi (24 stations); **(d)** Hainan (20 stations); **(e)** Guangdong (113 stations); **(f)** Fujian (13 stations); **(g)** Zhejiang (60 stations); **(h)** Taiwan/Republic of China (ROC) (72 stations); and **(i)** Hong Kong Special Administrative Region (SAR) (12 stations). O_3 measurements are available from April to July 2014. The model bias (NMBF) and correlation (r^2) between modelled and measured values are given at the top of the righthand figures. Simulated values are shown for three model simulations: without fire emissions (WRFChem_nofire); with FINN fire emissions (WRFChem_FINN); and with FINN emissions scaled upwards by a factor 1.5 (WRFChem_FINNx1.5).

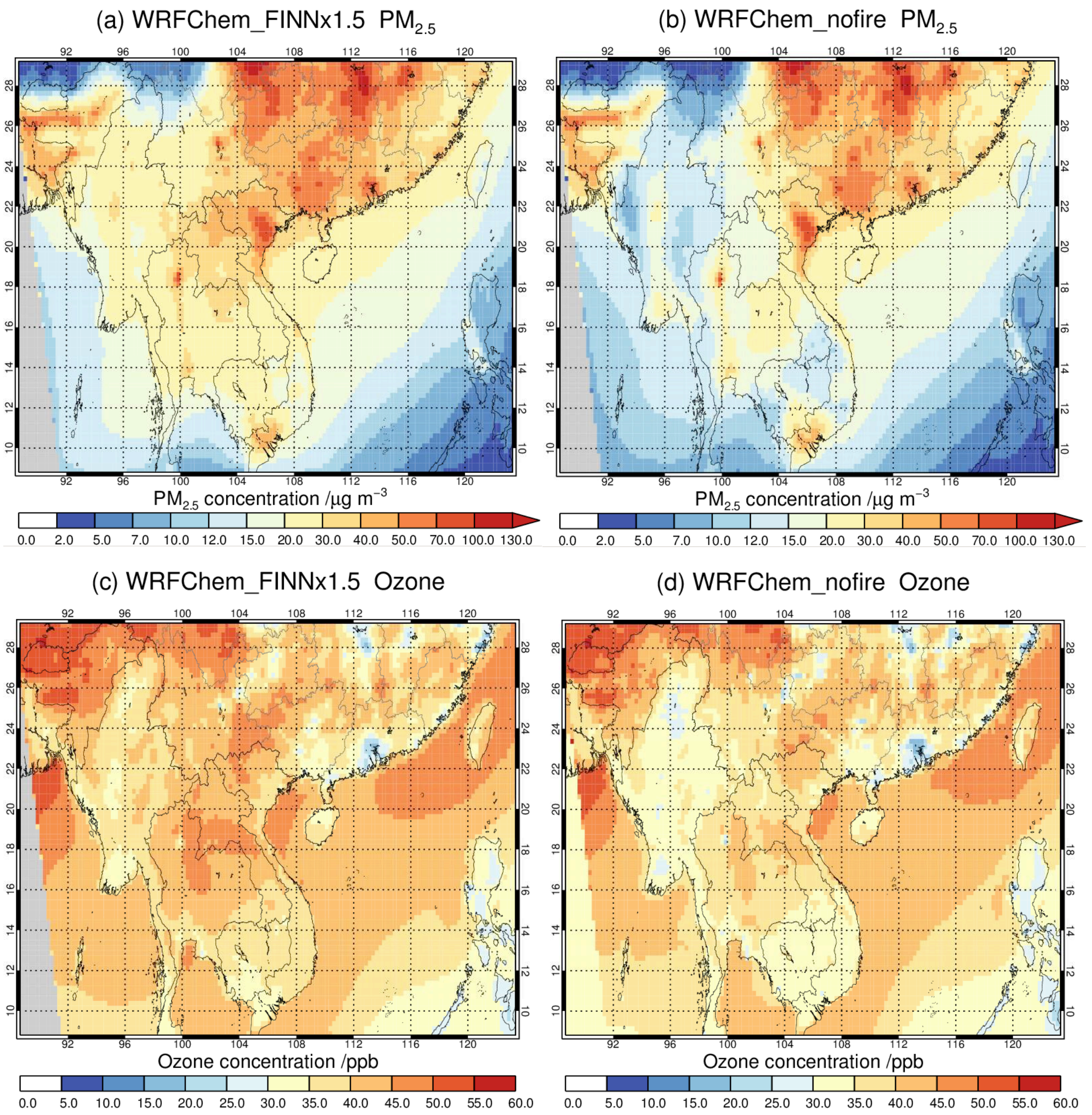

Figure S7. Spatial distribution of WRF-Chem-simulated annual mean surface **(a)** $PM_{2.5}$ and **(c)** ozone concentrations across Southeast Asia for 2014. Simulated concentrations are shown for the model simulation with FINN emissions scaled upwards by a factor 1.5 (WRFChem_FINNx1.5) in (a) and (c), and the model simulation without fire emissions (WRFChem_nofire) in **(b)** and **(d)**. Regions in grey are outside the model domain.

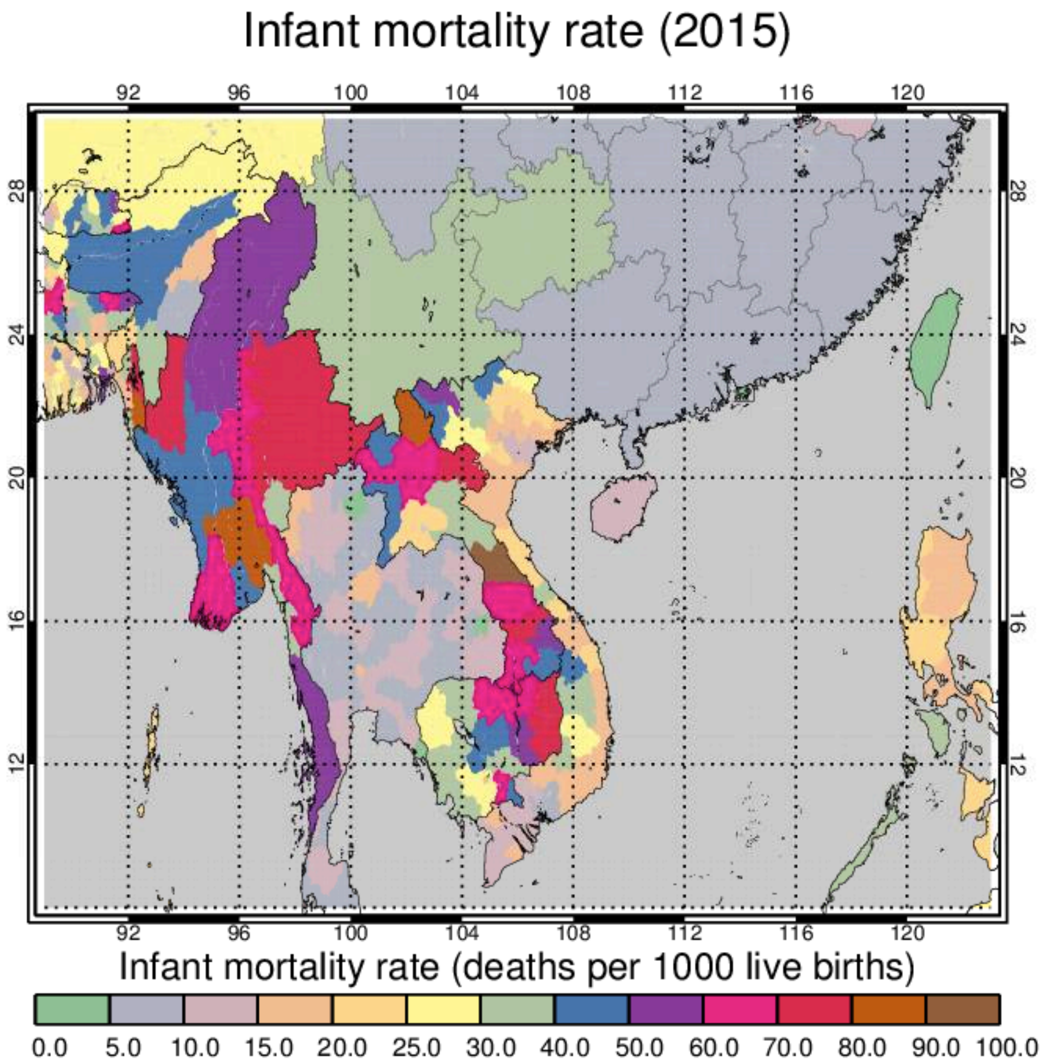

Figure S8. Spatial distribution of subnational infant mortality rate (IMR) estimates across Southeast Asia for the year of 2015 (CIESIN, 2018a). The gridded IMR estimates are at a spatial resolution of 30 arc-seconds (~1 km). The IMR for a region or country is defined as the number of children who die before their first birthday for every 1,000 live births.

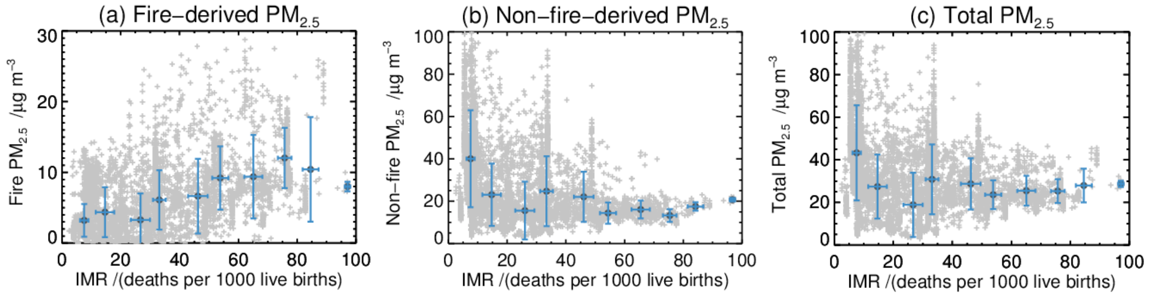

Figure S9. Gridded subnational Infant Mortality Rate (IMR; CIESIN, 2018a) values versus WRF-Chem simulated annual mean **(a)** fire-derived $PM_{2.5}$, **(b)** non-fire-derived $PM_{2.5}$, and **(c)** total $PM_{2.5}$ concentrations across the Southeast Asian domain. The blue data points show mean values for binned IMR data (bin size = 10 deaths per 1,000 live births); error bars show the standard deviation. The grey data points show all 0.25°x0.25° grid cell values across the domain.

References

Arnold, S. R., Chipperfield, M. P., and Blitz, M. A.: A three dimensional model study of the effect of new temperature dependent quantum yields for acetone photolysis, J. Geophys. Res., 110, D22305, doi:10.1029/2005JD005998, 2005.

Balk, D., G. D. Deane, M. A. Levy, A. Storeygard, and S. Ahamed. 2006. The Biophysical Determinants of Global Poverty: Insights from an Analysis of Spatially Explicit Data. Paper presented at the 2006 Annual Meeting of the Population Association of America, Los Angeles, CA, 30 March-1 April 2006.

Burnett, R., Chen, H., Szyszkowicz, M., Fann, N., Hubbell, B., Pope Iii, C.A., Apte, J.S., Brauer, M., Cohen, A., Weichenthal, S., Coggins, J., Di, Q., Brunekreef, B., Frostad, J., Lim, S.S., Kan, H., Walker, K.D., Thurston, G.D., Hayes, R.B., Lim, C.C., Turner, M.C., Jerrett, M., Krewski, D., Gapstur, S.M., Diver, W.R., Ostro, B., Goldberg, D., Crouse, D.L., Martin, R.V., Peters, P., Pinault, L., Tjepkema, M., van Donkelaar, A., Villeneuve, P.J., Miller, A.B., Yin, P., Zhou, M., Wang, L., Janssen, N.A.H., Marra, M., Atkinson, R.W., Tsang, H., Quoc Thach, T., Cannon, J.B., Allen, R.T., Hart, J.E., Laden, F., Cesaroni, G., Forastiere, F., Weinmayr, G., Jaensch, A., Nagel, G., Concin, H., and Spadaro, JV.: Global estimates of mortality associated with longterm exposure to outdoor fine particulate matter, Proc. Natl. Acad. Sci. U.S.A., 115 (38), 9592-9597, doi: 10.1073/pnas.1803222115, http://www.pnas.org/content/pnas/115/38/9592.full.pdf, 2018.

Chipperfield, M. P.: New version of the TOMCAT/SLIMCAT offline chemical transport model: Intercomparison of stratospheric tracer experiments, Q. J. Roy. Meteor. Soc., 132, 1179–1203, 2006.

CIESIN (Center for International Earth Science Information Network), Columbia University. Gridded Population of the World, Version 4 (GPWv4): Population Count. Palisades, NY: NASA Socioeconomic Data and Applications Center (SEDAC). https://doi.org/10.7927/H4X63JVC, 2016a.

CIESIN (Center for International Earth Science Information Network), Columbia University. Gridded Population of the World, Version 4 (GPWv4): Land and Water Area. Palisades, NY: NASA Socioeconomic Data and Applications Center (SEDAC). https://doi.org/10.7927/H45M63M9, 2016b.

CIESIN (Center for International Earth Science Information Network), Columbia University. Global Subnational Infant Mortality Rates, Version 2. Palisades, NY: NASA Socioeconomic Data and Applications Center (SEDAC). https://doi.org/10.7927/H4PN93JJ, 2018a. Accessed: 2 November 2020.

CIESIN (Center for International Earth Science Information Network), Columbia University. Documentation for the Global Subnational Infant Mortality Rates, Version 2. Palisades, NY: NASA Socioeconomic Data and Applications Center (SEDAC). https://doi.org/10.7927/H44J0C25, 2018b.

Dee, D.P., Uppala, S.M., Simmons, A.J., Berrisford, P., Poli, P., Kobayashi, S., Andrae, U., Balmaseda, M.A., Balsamo, G., Bauer, P., Bechtold, P., Beljaars, A.C.M., van de

Berg, L., Bidlot, J., Bormann, N., Delsol, C., Dragani, R., Fuentes, M., Geer, A.J., Haimberger, L., Healy, S.B., Hersbach, H., Hólm, E.V., Isaksen, L., Kållberg, P., Köhler, M., Matricardi, M., McNally, A.P., Monge-Sanz, B.M., Morcrette, J.-J., Park, B.-K., Peubey, C., de Rosnay, P., Tavolato, C., Thépaut, J.-N. and Vitart, F. (2011), The ERA-Interim reanalysis: configuration and performance of the data assimilation system. Q.J.R. Meteorol. Soc., 137: 553-597. https://doi.org/10.1002/qj.828

Dentener, F., Kinne, S., Bond, T., Boucher, O., Cofala, J., Generoso,S., Ginoux, P., Gong, S., Hoelzemann, J. J., Ito, A., Marelli, L.,Penner, J. E., Putaud, J.-P., Textor, C., Schulz, M., van der Werf,G. R., and Wilson, J.: Emissions of primary aerosol and precur-sor gases in the years 2000 and 1750 prescribed data-sets for Ae-roCom, Atmos. Chem. Phys., 6, 4321–4344, doi:10.5194/acp-6-4321-2006, 2006.

Emmons, L. K., Walters, S., Hess, P. G., Lamarque, J.-F., Pfister, G. G., Fillmore, D., Granier, C., Guenther, A., Kinnison, D., Laepple, T., Orlando, J., Tie, X., Tyndall, G., Wiedinmyer, C., Baughcum, S. L., and Kloster, S.: Description and evaluation of the Model for Ozone and Related chemical Tracers, version 4 (MOZART-4), Geosci. Model Dev., 3, 43-67, https://doi.org/10.5194/gmd-3-43-2010, 2010.

GBD (Global Burden of Disease) 2015 Risk Factors Collaborators: Global, regional, and national comparative risk assessment of 79 behavioural, environmental and occupational, and metabolic risks or clusters of risks, 1990-2015: a systematic analysis for the Global Burden of Disease Study 2015, Lancet, 388, 1659–1724, https://doi.org/10.1016/S0140-6736(16)31679-8, 2016.

GBD (Global Burden of Disease) 2017 Risk Factors Collaborators: Global, regional, and national comparative risk assessment of 84 behavioural, environmental and occupational, and metabolic risks or clusters of risks for 195 countries and territories, 1990–2017: a systematic analysis for the Global Burden of Disease Study 2017, Lancet, 392, 1923–1994, DOI:https://doi.org/10.1016/S0140-6736(18)32225-6, 2018.

Granier, C., Bessagnet, B., Bond, T., D'Angiola, A., Denier van derGon, H., Frost, G. J., Heil, A., Kaiser, J. W., Kinne, S., Klimont,Z., Kloster, S., Lamarque, J.-F., Liousse, C., Masui, T., Meleux, F., Mieville, A., Ohara, T., Raut, J.-C., Riahi, K., Schultz, M.G., Smith, S. J., Thompson, A., Aardenne, J., van der Werf, G.R., and Vuuren, D. P.: Evolution of anthropogenic and biomass burning emissions of air pollutants at global and regional scales during the 1980–2010 period, Climatic Change, 109, 163–190, 2011.

Guenther, A., Hewitt, C. N., Erickson, D., Fall, R., Geron, C.,Graedel, T., Harley, P., Klinger, L., Lerdau, M., McKay, W. A.,Pierce, T., Scholes, B., Steinbrecher, R., Tallamraju, R., Taylor, J., and Zimmerman, P.: A global model of natural volatile organic compound emissions, J. Geophys. Res., 100, 8873–8892, 1995.

Guenther, A., Karl, T., Harley, P., Wiedinmyer, C., Palmer, P. I., and Geron, C.: Estimates of global terrestrial isoprene emissions using MEGAN (Model of Emissions of Gases and Aerosols from Nature), Atmos. Chem. Phys., 6, 3181-3210, https://doi.org/10.5194/acp-6-3181-2006, 2006.

Hijmans, R., University of California, Berkeley Museum of Vertebrate Zoology, Kapoor, J., Wieczorek, J., International Rice Research Institute, et al.: Global Administrative Areas (GADM): Boundaries without limits. Version 2.8, 2016.

Hodzic, A. and Jimenez, J. L.: Modeling anthropogenically controlled secondary organic aerosols in a megacity: a simplified framework for global and climate models. Geosci. Model Dev., 4, 901-917, 2011.

Hodzic, A. and Knote, C.: WRF-Chem 3.6.1: MOZART gas-phase chemistry with MOSAIC aerosols, Atmospheric Chemistry Division (ACD), National Center for Atmospheric Research (NCAR), 2014.

Institute for Health Metrics and Evaluation: Global Burden of Disease (GBD) Compare Data Visualization. Retrieved February 13, 2019, from https://vizhub.healthdata.org/gbd-compare/, 2019.

Janssens-Maenhout, G., Crippa, M., Guizzardi, D., Dentener, F., Muntean, M., Pouliot, G., Keating, T., Zhang, Q., Kurokawa, J., Wankmüller, R., Denier van der Gon, H., Kuenen, J. J. P., Klimont, Z., Frost, G., Darras, S., Koffi, B., and Li, M.: HTAP_v2.2: a mosaic of regional and global emission grid maps for 2008 and 2010 to study hemispheric transport of air pollution, Atmos. Chem. Phys., 15, 11411-11432, https://doi.org/10.5194/acp-15-11411-2015, 2015.

Knote, C., Hodzic, A., Jimenez, J. L., Volkamer, R., Orlando, J. J., Baidar, S., Brioude, J., Fast, J., Gentner, D. R., Goldstein, A. H., Hayes, P. L., Knighton, W. B., Oetjen, H., Setyan, A., Stark, H., Thalman, R., Tyndall, G., Washenfelder, R., Waxman, E., and Zhang, Q.: Simulation of semi-explicit mechanisms of SOA formation from glyoxal in aerosol in a 3-D model, Atmos. Chem. Phys., 14, 6213-6239, https://doi.org/10.5194/acp-14-6213-2014, 2014.

LeGrand, S. L., Polashenski, C., Letcher, T. W., Creighton, G. A., Peckham, S. E., and Cetola, J. D.: The AFWA dust emission scheme for the GOCART aerosol model in WRF-Chem v3.8.1, Geosci. Model Dev., 12, 131-166, https://doi.org/10.5194/gmd-12-131-2019, 2019.

Mann, G. W., Carslaw, K. S., Spracklen, D. V., Ridley, D. A., Manktelow, P. T., Chipperfield, M. P., Pickering, S. J., and Johnson, C. E.: Description and evaluation of GLOMAP-mode: a modal global aerosol microphysics model for the UKCA composition-climate model, Geosci. Model Dev., 3, 519-551, doi:10.5194/gmd-3-519-2010, 2010.

NCAR (National Center for Atmospheric Research): ACOM MOZART-4/GEOS-5 global model output. Available at: http://www.acom.ucar.edu/wrf-chem/mozart.shtml UCAR, 2016.

NCEP: National Weather Service, NOAA & U.S. Department of Commerce. NCEP Final (FNL) Operational Model Global Tropospheric Analyses, continuing from July 1999. Research Data Archive at the National Center for Atmospheric Research, Computational and Information Systems Laboratory, https://doi.org/10.5065/D6M043C6, 2000.

NCEP: National Weather Service, NOAA & U.S. Department of Commerce. NCEP Global Forecast System (GFS) Analyses and Forecasts. Research Data Archive at the National Center for Atmospheric Research, Computational and Information Systems Laboratory, http://rda.ucar.edu/datasets/ds084.6/, 2007.

Rohde, R. A. and Muller, R. A.: Air pollution in China: mapping of concentrations and sources, PLoS ONE, 10, 1-14, doi:10.1371/journal.pone.0135749, 2015.

Scott, C. E., Rap, A., Spracklen, D. V., Forster, P. M., Carslaw, K. S., Mann, G. W., Pringle, K. J., Kivekäs, N., Kulmala, M., Lihavainen, H., and Tunved, P.: The direct and indirect radiative effects of biogenic secondary organic aerosol, Atmos. Chem. Phys., 14, 447-470, https://doi.org/10.5194/acp-14-447-2014, 2014.

Spracklen, D. V., Pringle, K. J., Carslaw, K. S., Chipperfield, M. P., and Mann, G. W.: A global off-line model of size-resolved aerosol microphysics: I. Model development and prediction of aerosol properties, Atmos. Chem. Phys., 5, 2227-2252, doi:10.5194/acp-5-2227-2005, 2005.

Turner, M. C., Jerrett, M., Pope III, C. A., Krewski, D., Gapstur, S. M., Diver, R. W., et al.: Long-Term Ozone Exposure and Mortality in a Large Prospective Study. American Journal of Respiratory and Critical Care Medicine, 193(10), 1134–1142. https://doi.org/10.1164/rccm.201508-1633OC, 2016.

United Nations Inter-agency Group for Child Mortality Estimation: Infant mortality rate estimates developed by the United Nations Inter-agency Group for Child Mortality Estimation (UNIGME), 2020. Available at www.childmortality.org. Accessed: 5 May 2021.

World Health Organization (WHO): Ambient Air Pollution: A Global Assessment Of Exposure And Burden Of Disease, Vol. 121, World Health Organization, Geneva, 1–131, 2016.

World Health Organization (WHO): WHO Global Ambient Air Quality Database (update 2018) edition, Version 1.0, Geneva, World Health Organization, 2018. Available at: https://www.who.int/airpollution/data/cities/en/, last access: 1 August 2019.

Yin, P., Brauer, M., Cohen, A., Burnett, R. T., Liu, J., Liu, Y., et al.: Long-term fine particulate matter exposure and nonaccidental and cause-specific mortality in a large national cohort of Chinese men. Environmental Health Perspectives, *125*(11), 117002-1-117002–117011. https://doi.org/10.1289/EHP1673, 2017.

Zaveri, R. A., Easter, R. C., Fast, J. D. and Peters, L. K.: Model for Simulating Aerosol Interactions and Chemistry (MOSAIC), J. Geophys. Res. Atmos., 113, D13204, doi:10.1029/2007JD008782, https://doi.org/10.1029/2007JD008782, 2008.
